# Gold nanoclusters elicit homeostatic perturbations in glioblastoma cells and adaptive changes of lysosomes

**DOI:** 10.7150/thno.37674

**Published:** 2020-01-01

**Authors:** Dusica Maysinger, Evan R. Gran, Franck Bertorelle, Hussein Fakhouri, Rodolphe Antoine, Esha S. Kaul, Dana M. Samhadaneh, Ursula Stochaj

**Affiliations:** 1 McGill University, Pharmacology & Therapeutics, Montreal, Canada; 2 Université Lyon, CNRS, Institut Lumière Matière, Lyon, France; 3 McGill University, Physiology, Montreal, Canada

**Keywords:** nanomaterials, cell organelle, organellar pH, lysosome positioning, proteostasis, cellular stress response

## Abstract

Unique physicochemical features place gold nanoclusters at the forefront of nanotechnology for biological and biomedical applications. To date, information on the interactions of gold nanoclusters with biological macromolecules is limited and restricts their use in living cells.

**Methods**: Our multidisciplinary study begins to fill the current knowledge gap by focusing on lysosomes and associated biological pathways in U251N human glioblastoma cells. We concentrated on lysosomes, because they are the intracellular destination for many nanoparticles, regulate cellular homeostasis and control cell survival.

**Results**: Quantitative data presented here show that gold nanoclusters (with 15 and 25 gold atoms), surface-modified with glutathione or PEG, did not diminish cell viability at concentrations ≤1 µM. However, even at sublethal concentrations, gold nanoclusters modulated the abundance, positioning, pH and enzymatic activities of lysosomes. Gold nanoclusters also affected other aspects of cellular homeostasis. Specifically, they stimulated the transient nuclear accumulation of TFEB and Nrf2, transcription factors that promote lysosome biogenesis and stress responses. Moreover, gold nanoclusters also altered the formation of protein aggregates in the cytoplasm. The cellular responses elicited by gold nanoclusters were largely reversible within a 24-hour period.

**Conclusions**: Taken together, this study explores the subcellular and molecular effects induced by gold nanoclusters and shows their effectiveness to regulate lysosome biology. Our results indicate that gold nanoclusters cause homeostatic perturbations without marked cell loss. Notably, cells adapt to the challenge inflicted by gold nanoclusters. These new insights provide a framework for the further development of gold nanocluster-based applications in biological sciences.

## Introduction

The unique physical and chemical properties of gold nanoclusters (AuNCs) make them attractive nanostructures for diverse applications. In addition to imaging, various functionalized AuNCs have been used as biosensors, diagnostic tools or nanocarriers for drugs 1-4. Some AuNCs have catalytic properties that are potentially useful for biomedical investigations 5.

AuNCs described so far contain between 11 and more than 270 gold atoms (reviewed in 6). AuNCs with 25 gold atoms stand out, because they are stable, easy to prepare, and methods for their functionalization are well established. A variety of functionalization approaches to modify AuNC surfaces have been used to enhance their dispersibility, solubility, and minimize or prevent aggregation (reviewed in 7, 8). AuNC modifications play an essential role for *in vivo* experiments, because gold atoms without adequate ligand protection can act as nanozymes and change the intracellular redox status 9, 10.

A common functionalization strategy is ligand exchange under conditions that are appropriate for the selected ligand and AuNC 3, 11. These protocols have been applied to produce thiol-stabilized AuNCs that frequently contain glutathione (GSH). Such AuNCs have been used for bioimaging in cells and *in vivo* 2. To date, in-depth studies on AuNCs were mostly performed *in vitro* using cell-free model systems.

Following cellular uptake, most nanomaterials will locate to lysosomes 12, 13. These membrane-delimited organelles maintain cellular homeostasis through the degradation of damaged organelles, misfolded proteins and internalized exogenous particles 14-16. Lysosomes also sense the cellular nutrient status, respond to stress and exocytose macromolecular material 14, 17-19.

Lysosomal activities are controlled on multiple levels. The activities of lysosomal enzymes, including various proteases, depend on the low pH of the organelle. Furthermore, lysosome biogenesis is regulated by transcription factors EB (TFEB) and E3 (TFE3) 17, 20. TFEB and TFE3, upon translocation into the nucleus, promote the expression of genes that stimulate lysosome biogenesis. Aside from lysosome abundance, their positioning within the cell is also critical, because lysosomal pH and enzymatic activities are determined by the organelle location 21, 22. Specifically, lysosomes adjacent to the nucleus are characterized by a more acidic pH, whereas organelles closer to the cell periphery are less acidic. As lysosomes control a multitude of cellular processes, their dysfunction has been associated with cancer, neurological or metabolic disorders 15, 19.

Nanomaterials can alter different aspects of cell physiology, and they may elicit stress responses 23-25. Such stress-induced changes are exemplified by the nuclear translocation of the transcription factor Nrf2 (nuclear factor erythroid 2-related factor 2, NFE2L2) 26, 27 and the formation of cytoplasmic stress granules 28-30. While Nrf2 helps to restore redox homeostasis through the expression of antioxidant-related genes 31, stress granule formation promotes cell survival under harmful growth conditions 28-30.

In previous studies, AuNCs were reported to have low toxicity in glioblastoma cells 32. However, the impact of AuNCs on cell processes remain largely unknown 4, and sub-lethal effects or adaptive responses have not been defined.

For the work described here, we selected AuNCs with 15 or 25 gold atoms functionalized with glutathione (GSH) or polyethylene glycol (PEG) to evaluate their impact on organelles and other subcellular compartments in glioblastoma cells. Our focus was on lysosomes and cellular stress responses, because they provide a measurable readout for nanoparticle-induced effects on cell physiology. Collectively, the presented studies suggest that in glioblastoma cells AuNCs prompt the adaption of lysosomal properties and stress-responsive pathways. The characterization of these processes at the cellular and molecular level is crucial for the further development of AuNC-based theranostics.

## Results and discussion

### Synthesis and characterization of gold nanoclusters

**Figure 1:**
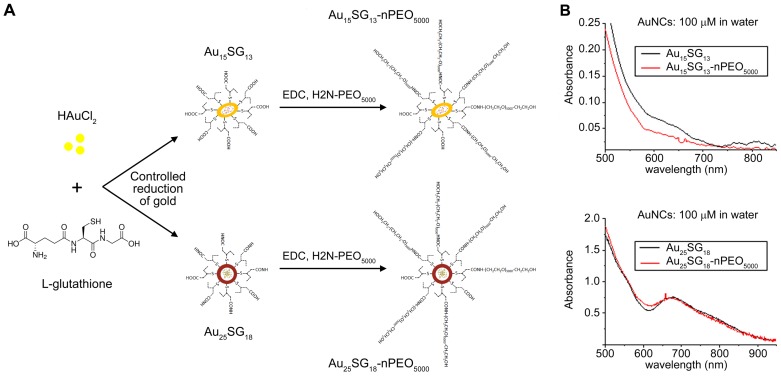
Synthesis and UV-vis absorption spectra of AuNCs. (A) Schematic representation of AuNC synthesis. AuNCs surfaces were modified with glutathione (SG) and PEG. Following the controlled reduction of gold with L-glutathione, AuNCs were PEGylated using the peptide coupling method described in the Methods section. (B) Absorbance spectra of Au_15_ and Au_25_ nanoclusters. Absorbance spectra for the different AuNCs indicated were obtained at a final concentration of 100 µM in water.

Glutathione-protected AuNCs were synthesised via a controlled reduction of gold (see Methods section). The monodispersity of gold cluster sizes (Au_15_SG_13_ and Au_25_SG_18_) was verified by ESI-mass spectrometry. The PEGylated AuNCs were prepared by covalent peptide coupling of PEG_5000_-NH_2_ to the surface carboxylic acid groups of GSH (Figure 1A). Figure 1B shows the UV-vis absorption spectra in solution of the synthesized AuNCs. The main features of spectra remain unchanged by post-covalent peptide coupling of PEG_5000_-NH_2_. However, the fluorescence intensity is strongly enhanced for PEGylated AuNCs as compared to unmodified AuSG nanoclusters, in particular for Au_15_NCs. This enhancement may be caused by the reduced solvent accessibility as well as “rigidification” of the ligand shell upon addition of PEG on the surface of AuNCs 33, 34. The average hydrodynamic diameter of the resulting AuSG nanoclusters and PEGylated AuSG nanoclusters was measured by time-resolved fluorescence anisotropy techniques, which are accurate sizing techniques for fluorescent NCs 35-38. An increase of ~3 nm in the size of PEGylated Au_15_SG_13_ (Au_15_PEG) was observed and can be contributed to the extended hydration layer thickness due to the polymer chain of PEG (Table S1).

### Effect of gold nanoclusters on glioblastoma cell viability

**Figure 2:**
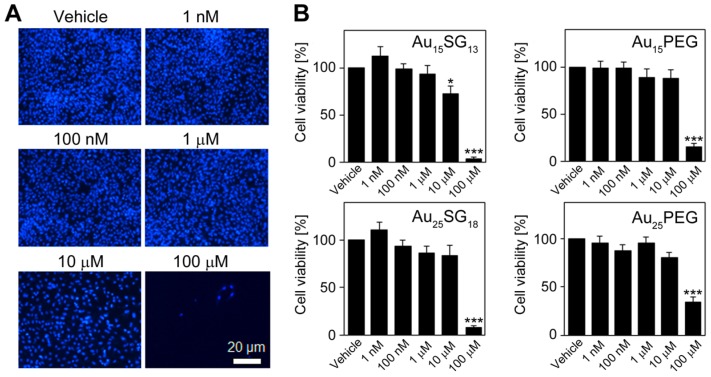
Impact of AuNCs on cell numbers. (A) U251N cells were treated with Au_15_SG_13_ for 72 hours with the concentrations indicated. Cells were identified with Hoechst 33342 staining of the nuclei as described in the Methods section. (B) Upon incubation with different AuNCs, cell numbers were quantified and results were normalized to vehicle controls. Graphs depict the average ± SEM for three independent experiments. Significant differences between the vehicle control and AuNC-treated cells are marked. *, *p*<0.05; ***, *p*<0.001.

The physiological responses of living human glioblastoma cells to the AuNCs illustrated in Figure 1 are currently unknown. To address this point, we examined how these AuNCs affect U251N glioblastoma cell viability, lysosomal properties and functions relevant to homeostatic and cytoskeletal biomarkers. We first assessed the viability of glioblastoma cells exposed to AuNCs. Quantitative data for concentration-dependent effects on cell viability were obtained by two complementary methods: by cell number counts (Figure 2) and measurements of metabolic activity (Figure S1).

For all AuNCs tested, cell numbers declined significantly at a final concentration of 100 μM (*p*<0.001). At 10 μM, only Au_15_SG_13_ diminished cell numbers; this effect was moderate but significant (reduction by ~25%). No significant changes were observed when AuNCs were present at 1 μM or lower concentrations (Figure 2). The impact of AuNCs was examined independently by the measurement of metabolic activity (MTT assay; Figure S1). Together, both methods indicated that increasing AuNC concentrations reduced U251N cell number and metabolic activity. However, at or below 1 µM no or only minor changes were observed (Figures 2, S1).

*In vivo* AuNCs are excreted to a large extent via renal clearance 39, 40. Therefore, we assessed AuNC toxicity in HEK293 cells which are derived from the human renal epithelium (Figure S1). At ≤1 µM, Au_15_SG_13_ had only moderate impact; metabolic activities remained ≥76% of the vehicle control. Moreover, Au_25_SG_18_ had no significant effect on the metabolic activity at any concentration tested.

Taken together, results in Figures 2 and S1 are consistent with unrelated studies by us that examined the impact of AuNCs on non-transformed neural cells 41. In dissociated and 3D organotypic cultures, Au_15_SG_13_ or Au_25_SG_18_ were not deleterious to neurons at final concentrations <10µM.

### Gold nanoclusters modulate the abundance of acidic compartments in glioblastoma cells

**Figure 3:**
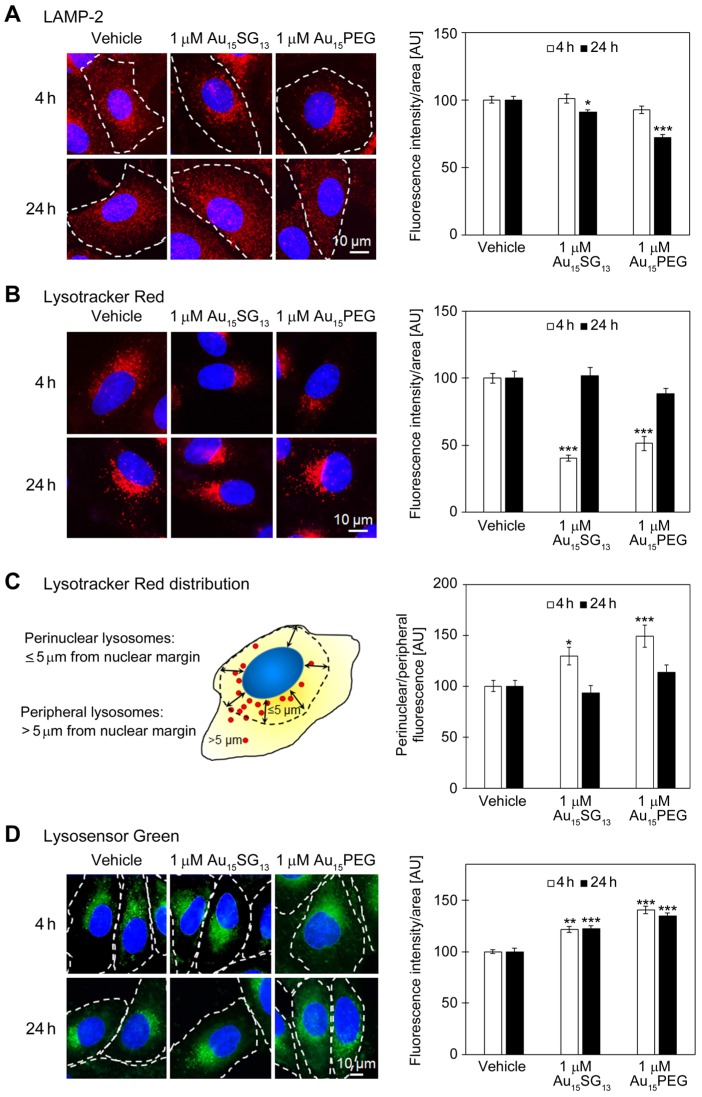
Evaluation of lysosomes in U251N cells. All graphs depict results normalized to the vehicle control; data are shown as average ± SEM; *p<0.05, **p<0.01, ***p<0.001; AU, arbitrary units. (A) Detection of LAMP-2. Cells were treated with 1 μM Au_15_SG_13_ or 1 μM Au_15_PEG for 4 or 24 hours. LAMP-2 was located by immunocytochemistry (red); nuclei were demarcated with Hoechst 33342 (blue). Fluorescence intensities/area were quantified for at least 97 cells per condition. (B) Lysosome staining with Lysotracker Red. Cells were incubated with vehicle, 1 μM Au_15_SG_13_ or 1 μM Au_15_PEG for 4 or 24 hours. The bars depict the average fluorescence intensity/area ± SEM; 52 to 134 cells were assessed per condition. (C) The distribution of Lysotracker Red signals was determined for the 4- and 24-hour treatment shown in part B. The fluorescence intensities were quantified for a 5-μm area adjacent to the nuclear margin (perinuclear) and for peripheral cell regions. The ratio of perinuclear/peripheral signals was calculated for 44 to 58 cells for each condition. Results are depicted as average ± SEM. (D) Staining of U251N cells with Lysosensor Green. U251N cells treated with 1 μM Au_15_SG_13_ or 1 μM Au_15_PEG for 4 or 24 hours were incubated with Lysosensor Green and imaged as described in the Methods section. Graphs depict average fluorescence intensities per area ± SEM; measurements were performed for a minimum of 81 cells per condition and at least two independent experiments.

Many internalized nanoparticles accumulate in lysosomes, where they may alter organellar properties. A set of experiments was carried out to evaluate specific aspects of lysosome biology using different tools (Figure 3). The abundance of lysosomes was monitored with the membrane protein LAMP-2, a marker for lysosomes and late endosomes 42. LysoTracker® Red DND-99 (in the following called Lysotracker Red) is an established fluorescent probe to identify and quantify acidic vesicles 43. We also assessed the subcellular lysosome location, because lysosomes close to the nucleus are characterized by a lower pH and enhanced activities of lysosomal enzymes 21, 22, 44. LysoSensor™ DND-189 (here called Lysosensor Green) was used to examine the pH of acidic organelles 45. We confirmed that Lysotracker Red and Lysosensor Green are appropriate tools for our study; their fluorescence emission at pH 7.2 or pH 4.5 was not markedly altered by AuNCs (Figures S2, S3).

The abundance of LAMP-2 positive vesicles was not significantly changed after 4 hours (Figure 3A), but moderately reduced at 24 hours, both for Au_15_SG_13_ (91% of vehicle control) and Au_15_PEG (72% of vehicle control). These results are consistent with the model that U251N cells can adapt to AuNCs without cell loss (Figure 2). Since LAMP-2 is located predominantly in lysosomal membranes, the data suggest an overall reduction in lysosomal size and/or number.

LAMP-2 immunostaining does not inform on the functional state of lysosomes. This information was obtained with fluorescent probes for lysosomes and cathepsin B assessment (Figure 3B-D and below). At 4 hours, the intensity of Lysotracker Red diminished significantly when compared to vehicle controls (100%), declining to 40% for Au_15_SG_13_ and 51% for Au_15_PEG. This decrease was transient only; after 24 hours Lysotracker Red fluorescence intensities were similar in cells treated with vehicle (100%), Au_15_SG_13_ (102%) or Au_15_PEG (88%).

The subcellular lysosome distribution is highly dynamic and linked to organellar pH 14, 44. Specifically, lysosomes located close to the nucleus are more acidic than their counterparts at the cell periphery. For both Au_15_SG_13_ and Au_15_PEG the perinuclear/peripheral ratio increased for Lysotracker Red at 4 hours (Figure 3C). Such a shift could be due to a rise in perinuclear fluorescence, loss in peripheral fluorescence, or a combination of both.

Remarkably, the distribution of Lysotracker Red-stained compartments was restored to vehicle controls after 24 hours. These data indicate that Au_15_SG_13_ and Au_15_PEG impaired the subcellular location of acidic vesicles only in the short term. Taken together, LAMP-2 staining, Lysotracker Red fluorescence intensity and distribution suggest adaptation of U251N cells within a 24-hour period of AuNC exposure.

The relocation of lysosomes towards the nucleus facilitates their acidification and enhances lysosome activities 44. To better define the AuNC-dependent effects on lysosome distribution, we performed single-cell analyses and sorted results into bins (Figure S4). This comprehensive evaluation revealed that (i) lysosomal positioning is dynamic in U251N cells. Between 4 and 24 hours the perinuclear/peripheral ratio increased for all samples, including the vehicle control. (ii) After 24 hours, the distribution of lysosomes was similar for vehicle and AuNC-treated samples. However, treatment with 1 µM Au_15_SG_13_ or 1 µM Au_15_PEG increased the variability, with perinuclear/peripheral ratios >>4 for some of the cells. This extent of perinuclear accumulation was not observed for lysosomes in vehicle controls. Collectively, single-cell analyses indicate that AuNCs generate cell populations that are more heterogeneous with respect to the characteristics of acidic vesicles.

Lysosensor Green has the highest pH sensitivity at its pK_a_ of 5.2. Lysosensor Green fluorescence emission is enhanced when the organellar pH approaches this value or the abundance of acidic compartments rises. Incubation with 1 µM Au_15_SG_13_ or 1 µM Au_15_PEG increased significantly the fluorescence intensities/area at 4 hours. Notably, these differences between vehicle controls and AuNC-treated cells persisted for 24 hours. This can be interpreted as an acidification of intracellular vesicles, increase in the number of acidic vesicles, or a combination of both.

Taken together, the different methods described in Figures 3 and S4 support the model that AuNCs altered the properties of intracellular acidic vesicles, in particular lysosomes. At 1 µM concentration, these changes were non-toxic and at least partially reversible.

Unlike other AuNCs examined in the current study, at 10 µM Au_15_SG_13_ reduced U251N cell numbers (Figure 2). To determine whether this was accompanied by augmented changes in lysosomal properties, Lysotracker Red fluorescence intensity, lysosome distribution, Lysosensor Green signals, and cathepsin B activity (see below) were assessed (Figure S5). Overall trends for 10 µM and 1 µM Au_15_SG_13_ were similar, but some of the effects were more pronounced with higher Au_15_SG_13_ concentrations. Our results suggest that lysosomal properties can be fine-tuned by selecting appropriate concentrations of Au_15_SG_13_.

**Figure 4:**
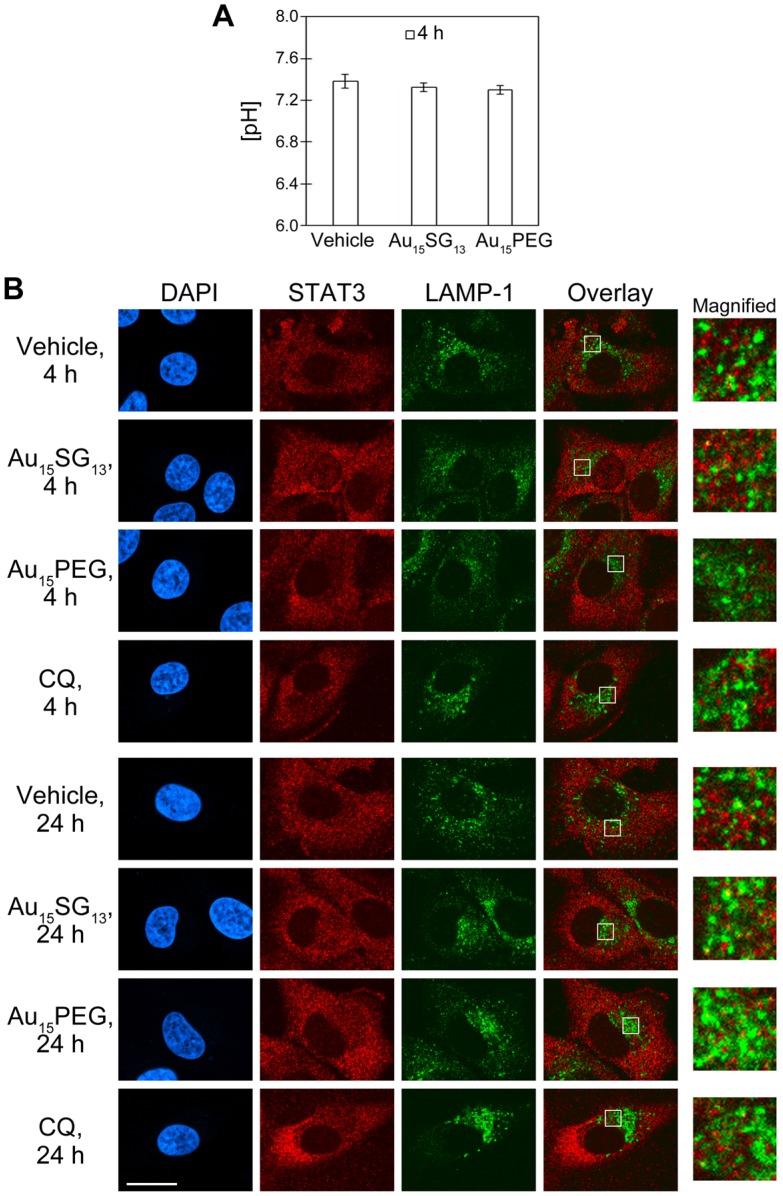
(A) Ratiometric measurement of cytoplasmic pH. U251N cells were treated for 4 hours with 1 µM Au_15_SG_13_ or 1 µM Au_15_PEG in serum-free medium. Following treatment, cells were incubated with 5 μM SNARF®-1 in serum-free DMEM for 45 min at 37^o^C. The pH values were determined by ratiometric fluorescence measurements and extrapolation from the calibration curve. The graph shows averages ± SEM for one experiment with triplicate samples. (B) Subcellular distribution of STAT3 and LAMP-1. U251N cells were incubated with vehicle, 1 µM Au_15_SG_13,_ 1 µM Au_15_PEG or 20 µM chloroquine (CQ) in serum-free medium for 4 hours or 24 hours as indicated. Cells were fixed and processed for immunocytochemistry as described in the Methods section. Scale bar is 20 µm. Selected regions of the overlay image were magnified 5-fold. Yellow color in the overlay images indicates co-localization of STAT3 and LAMP-1.

The changes of lysosomal distribution described in Figure 3 may suggest AuNC-dependent alterations in cellular pH homeostasis, in particular after a 4-hour incubation period. We addressed this point by monitoring possible effects on the cytoplasmic pH, using SNARF®-1 for ratiometric fluorescence measurements. Both 1 µM Au_15_SG_13_ and 1 µM Au_15_PEG caused a minor acidification of the cytoplasmic pH, but this amounted to less than 0.1 units (Figure 4A).

One of the mechanisms to stabilize the cytosolic pH relies on STAT3, a nuclear transcription factor that also associates with the cytoplasmic side of lysosomes 46. STAT3 interacts and stimulates v-ATPase activity. This interaction promotes lysosomal acidification and, at the same time, preserves a slightly basic cytosolic pH. In HeLa cells, approximately 5% of STAT3 co-purifies with lysosomes 46.

Figure 3D shows that Lysosensor Green signals increased after treatment with 1 μM Au_15_SG_13_ or 1 μM Au_15_PEG for 4 and 24 hours. This may indicate an enhanced acidification of lysosomes. STAT3 is a possible candidate protein to reduce the luminal pH of lysosomes. Therefore, we investigated whether STAT3 co-localized with lysosomes, using LAMP-1 as a marker for the lysosomal membrane. In Figure 4B, U251N cells were incubated with vehicle, 1 µM AuNCs or 20 µM chloroquine and fixed. Fixed cells were permeabilized with saponin to maintain the association of STAT3 with lysosomes. (Note that saponin does not efficiently permeabilize the nuclear envelope. Therefore, STAT3 was not detected in the nucleus.) Consistent with the Lysotracker Red distribution (Figure 3B) most of LAMP-1 was present in the vicinity of the nucleus (Figure 4B). By contrast, STAT3 distributed throughout the cytosol in a punctate fashion. This suggests the association of STAT3 with vesicles, which could be mediated through its interactions with various membrane proteins (e.g. EGFR) 47. However, co-localization with LAMP-1, which appears yellow in the overlay images, was rare under all conditions examined. Collectively, our results suggest that AuNCs did not induce marked changes to the stable association of STAT3 with lysosomes in U251N cells. This does not rule out a transient or weak interaction of STAT3 with lysosomal v-ATPase, which may have escaped detection.

### Au_15_SG_13_ increases the formation of F-actin, with minor changes in tubulin abundance and nuclear size

The intracellular distribution of lysosomes is regulated by the actin cytoskeleton and microtubules; both filamentous systems control different aspects of lysosomal movement 21, 22, 48. In particular, the interaction with filamentous actin (F-actin) modulates constrained diffusion, whereas microtubules and associated motors promote the directed movement of lysosomes 48.

**Figure 5:**
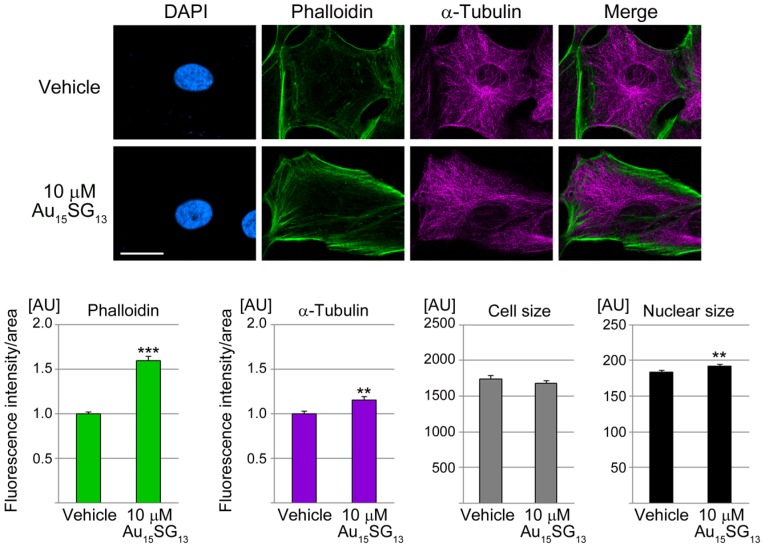
Au_15_SG_13_ increases the concentration of filamentous actin. U251N cells were incubated with 10 µM Au_15_SG_13_ for 24 hours, fixed and processed for immunohistochemistry to detect α-tubulin. F-actin was stained with Alexa Fluor® 488 phalloidin (Phalloidin); nuclei were demarcated with DAPI. Scale bar is 20 µm. Fluorescence intensities for phalloidin and α-tubulin were quantified for vehicle (105 cells) and Au_15_SG_13_ treated samples (126 cells). Graphs depict average + SEM for one representative experiment. Size was measured for 210 (vehicle) and 231 (Au_15_SG_13_) cells. Nuclear size was determined for 371 (vehicle) and 385 (Au_15_SG_13_) cells. AU, arbitrary units. Statistical evaluation was performed with Student's t-test; **, *p*<0.01; ***, *p*<0.001.

As described above, 10 µM Au_15_SG_13_ significantly altered cell numbers, metabolic activities (Figure 2, S1), and lysosomal characteristics (Figure S5). We selected this condition initially to examine cytoskeletal properties. After 24 hours, 10 µM Au_15_SG_13_ increased significantly the abundance of F-actin (Figure 5). Moreover, the formation of cortical F-actin at the cell periphery and of stress fibers was often enhanced. Adjacent to the nucleus cytoplasmic F-actin concentrations were elevated to 132% of the vehicle control (not shown), but this was not significant. At the same time, the abundance of α-tubulin increased slightly upon Au_15_SG_13_ treatment. However, no marked changes in microtubule organization were detected.

The actin cytoskeleton regulates cell volume and size 49, 50, and components of the cytoskeleton regulate nuclear size 51. While the size of U251N cells was not affected by 10 µM Au_15_SG_13_ after 24 hours (Figure 5), nuclei showed a minute increase in size.

Given the pronounced impact of 10 µM Au_15_SG_13_ on F-actin formation, we further examined U251N cells after incubation with 1 µM Au_15_SG_13_ or 1 µM Au_15_PEG. A 24-hour treatment with 1 µM AuNCs increased the F-actin content, cell and nuclear size, but the changes were small (Figure S6). Collectively, these results indicate that AuNCs reorganize the cytoskeleton in a concentration-dependent manner. We propose that the AuNC-induced effects on the cytoskeleton modulate the dynamic subcellular distribution of lysosomes and thereby the activities of lysosomal enzymes, such as cathepsin B (see below).

### Au_15_SG_13_ and Au_15_PEG induce transient TFEB nuclear accumulation and increase cathepsin B activity

**Figure 6:**
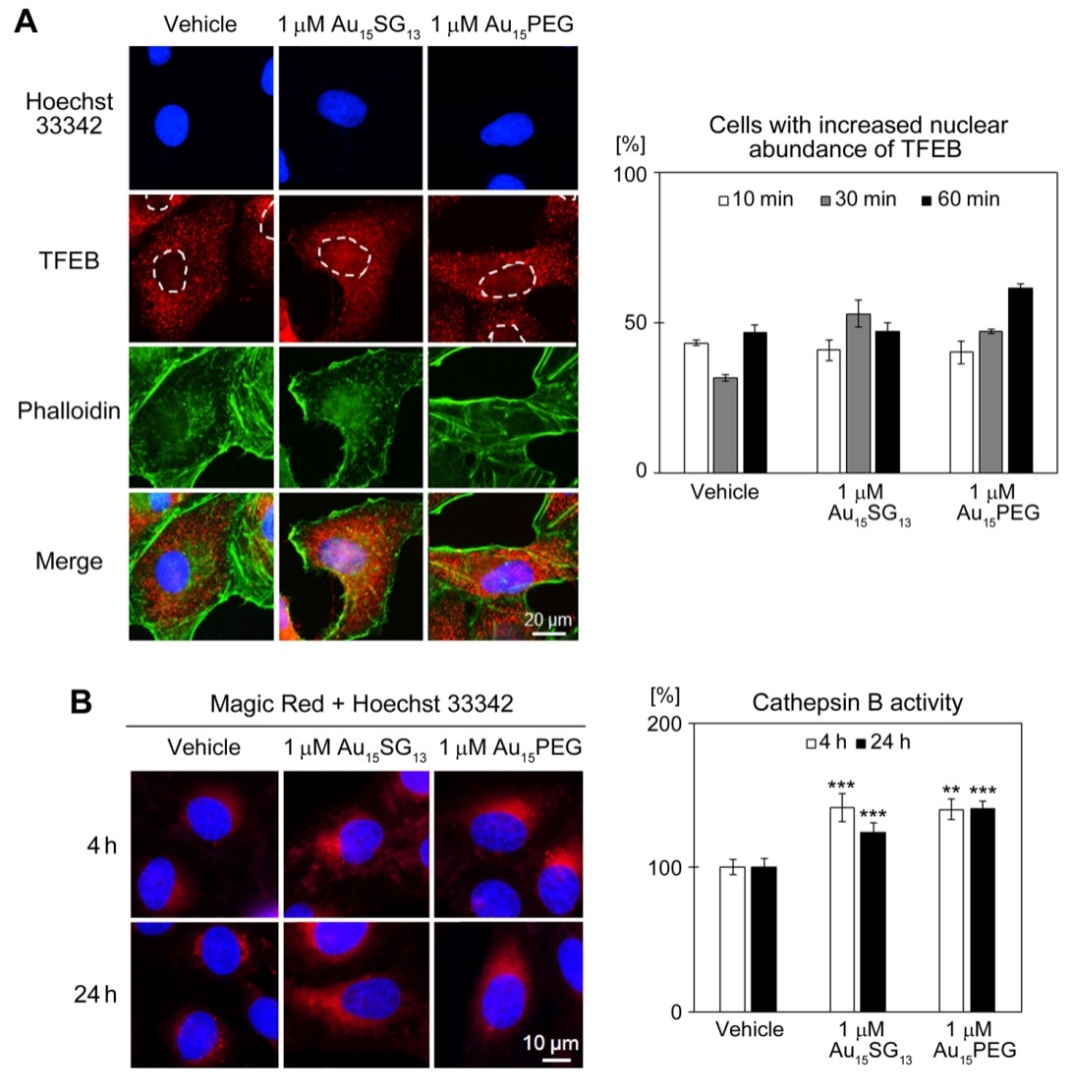
Effects of AuNCs on TFEB nucleocytoplasmic distribution and lysosomal activity. (A) U251N cells were treated with vehicle, 1 μM Au_15_SG_13_ or 1 μM Au_15_PEG in DMEM for the times indicated. Panels depict the results for cells incubated for 30 min with vehicle or AuNCs. TFEB was detected by immunocytochemistry, phalloidin stained F-actin, and Hoechst 33342 demarcated nuclei. Whole cell and nuclear fluorescence signals were quantified, and the nuclear/cytoplasmic ratio was calculated. The percentage of cells with elevated nuclear TFEB signals was determined for two independent experiments. For each condition and time point 80 to 101 cells were scored; bars show averages ± SEM. (B) AuNCs increase cathepsin B activity. Cathepsin B activity was measured in U251N cells treated with vehicle, 1 μM Au_15_SG_13_ or 1 μM Au_15_PEG. Cells were incubated with Magic Red as described in the Methods section. Hoechst 33342 identified nuclei. For quantification, Magic Red fluorescence intensity was normalized to the vehicle control. Bars depict average ± SEM for at least two independent experiments. Between 34 and 92 cells were analyzed per condition and time point; **, *p<*0.01; ***, *p<*0.001.

Results depicted in Figure 3 show an AuNC-dependent rise in acidic cellular compartments and a transient repositioning of lysosomes. These findings are consistent with an increase in lysosomal biogenesis 48, which was examined in Figure 6. Transcription factor EB (TFEB) regulates the expression of genes essential for lysosomal biogenesis and enzymatic activities, including the protease cathepsin B 17, 20, 52. TFEB also controls the positioning of lysosomes, stimulates their perinuclear accumulation and enhances lysosomal exocytosis 18, 19, 53.

Under normal growth conditions, TFEB resides predominantly in the cytosol. To upregulate lysosome biogenesis, TFEB concentrates in nuclei where it promotes the expression of multiple target genes 52. As compared to vehicle-treated samples, Au_15_SG_13_ and Au_15_PEG rapidly increased TFEB nuclear abundance (Figure 6A), albeit with somewhat different time courses. AuNC-induced nuclear accumulation of TFEB was reversible; it did not persist over a 24-hour period, when U251N cells were treated with 10 µM Au_15_SG_13_ (Figure S7). By contrast, Torin-1, a compound that concentrates TFEB in nuclei 54, led to sustained TFEB nuclear accumulation (Figure S7).

**Figure 7:**
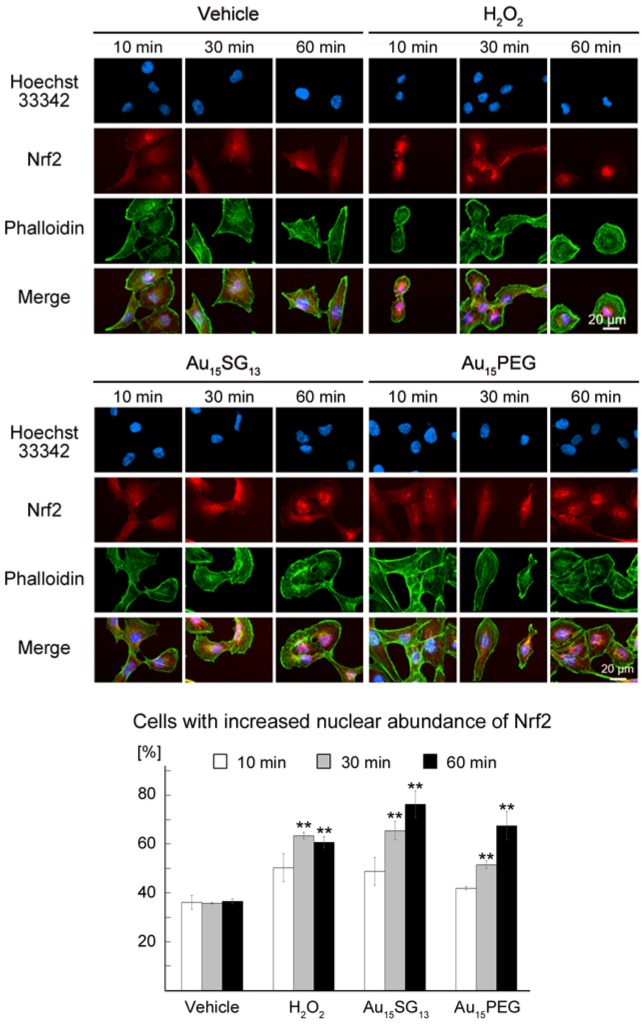
AuNCs promote the nuclear accumulation of Nrf2. U251N cells were treated for the times indicated with vehicle, 300 μM of H_2_O_2_, 10 μM of Au_15_SG_13_ or 10 μM Au_15_PEG for the times indicated. Cells were processed for immunocytochemistry with antibodies against Nrf2. Nuclei were detected with Hoechst 33342 and F-actin stained with phalloidin. After incubation for 10, 30 or 60 minutes, Nrf2 nuclear translocation was quantified by assessing Nrf2 fluorescence in the nucleus and cytosol. Three independent experiments were conducted and a total of 120 cells were analyzed. The graph shows average ± SEM; *, *p*<0.05; **, *p*< 0.01; ***, *p*<0.001.

The AuNC-dependent rise in acidic vesicles (Figure 3B-D) and the transient nuclear accumulation of TFEB (Figure 7A) could lead to the upregulation of lysosomal biogenesis and lysosomal enzyme activities. To test this model, we measured the activity of cathepsin B, a protease located in lysosomes. Incubation of U251N cells with 1 µM Au_15_SG_13_ or 1 µM Au_15_PEG increased significantly cathepsin B activity both at 4 and 24 hours. The AuNC-induced changes in cathepsin B activity (Figure 6B) correlated with enhanced Lysosensor Green fluorescence (Figure 3D). Moreover, the short-term rise in nuclear TFEB abundance is consistent with the increased perinuclear positioning of lysosomes after a 4-hour treatment with AuNCs (Figure 3C, 53).

Interestingly, despite the rise in TFEB abundance in the nucleus the signals for LAMP-2 were diminished (Figure 3A). One possible explanation for this phenomenon is lysosomal exocytosis, which reduces the intracellular concentrations of lysosomes; lysosomal exocytosis increases with the rise of TFEB abundance (see following section, 18, 19). Taken together, our results support the idea that AuNCs modulate cellular homeostasis, in part by stimulating processes that rely on lysosomal enzyme activities.

### Au_15_SG_13_ and Au_15_PEG transiently relocate the transcription factor Nrf2 to nuclei

TFEB nuclear accumulation can be accompanied by the activation of Nuclear factor (erythroid-derived 2)-like 2 (Nrf2) 55. The transcription factor Nrf2 is key to cellular homeostasis, as it regulates the expression of genes involved in the oxidative stress response. Under normal growth conditions, Nrf2 resides in the cytosol. However, the transcription factor translocates to the nucleus when the abundance of reactive oxygen species (ROS) increases 26.

Time-course experiments assessed the impact of AuNCs on Nrf2 location (Figure 7). Hydrogen peroxide (H_2_O_2_) provided a positive control; the oxidant activates Nrf2-dependent responses 56. Au_15_SG_13_ and Au_15_PEG elevated Nrf2 levels in the nucleus, especially in nucleoli. This nuclear relocation was time-dependent, and Nrf2 abundance in the nucleus was significantly increased at 30 and 60 minutes (Figure 7).

As described for TFEB (Figure 6A), AuNC-dependent nuclear accumulation of Nrf2 was only transient. After 24 hours incubation with 10 µM Au_15_SG_13_ Nrf2 abundance in nuclei was slightly reduced (Figure S8), but remained high when cells were exposed to 300 µM H_2_O_2_. Results for Nrf2 subcellular location further support the model that Au_15_SG_13_ and Au_15_PEG induce cellular stress, but cells recovered and adapted during a 24-hour incubation period. The changes in Nrf2 subcellular localization are consistent with the idea that AuNCs transiently elevated cellular ROS levels. While some AuNCs induce oxidative stress 57, the mechanisms through which ROS increase in Au_15_PEG-treated cells are currently not known.

### Au_15_SG_13_ nanoclusters reduce intracellular protein aggregates

**Figure 8:**
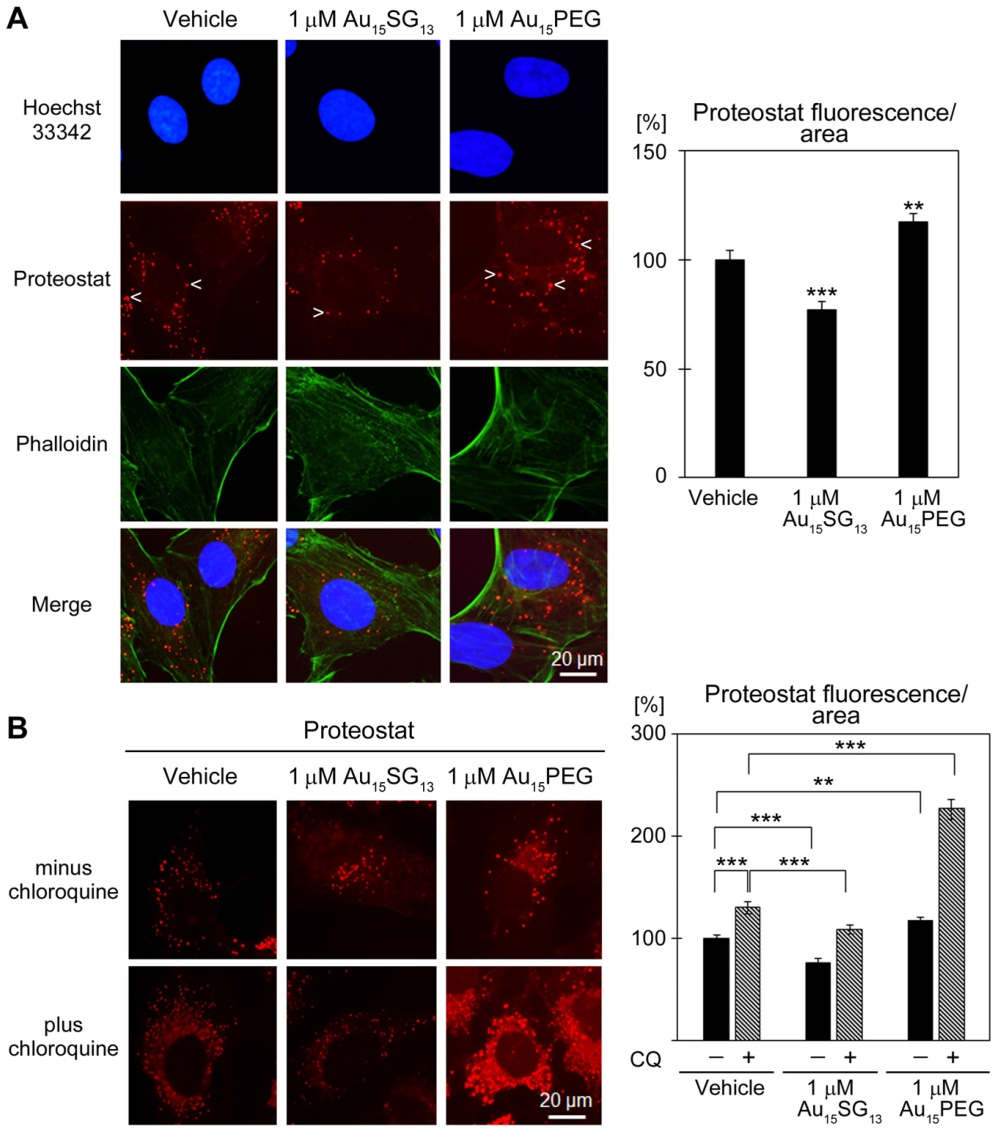
Effect of AuNCs on protein aggregation. (A) U251N cells treated with vehicle, 1 μM Au_15_SG_13_ or Au_15_PEG in DMEM for 24 hours were fixed and protein aggregation was evaluated with Proteostat. Some of the aggregates are marked with arrowheads. Fluorescence intensities/area were quantified for 97 to 105 cells for each condition. Results normalized to the vehicle control are shown as average ± SEM; ***, *p<*0.001. (B) U251N cells were incubated as described in part A. Chloroquine (CQ) was present during treatment as indicated. Proteostat signals were quantified for 88 to 105 cells per condition. Results were normalized to the vehicle control/minus chloroquine. Bars show average ± SEM; *p<0.05, **p<0.01, ***p<0.001.

Lysosomes are required to maintain protein homeostasis; they are essential to prevent the build-up of aggregated proteins 16. The Proteostat assay examined how AuNCs impinge on protein aggregation in the cytoplasm (Figure 8). After 24-hour treatment, protein aggregates were reduced by 1 μM Au_15_SG_13_ (78% of vehicle control), but increased with 1 μM Au_15_PEG (Figure 8A).

To further characterize the impact of 1 μM AuNCs on protein aggregate formation, we challenged U251N cells with chloroquine, a compound that disrupts lysosomal functions 58. Notably, in the presence of 20 µM chloroquine, protein aggregates were diminished with Au_15_SG_13_, and significantly enhanced by Au_15_PEG (Figure 8B).

These experiments revealed a striking difference between Au_15_SG_13_ and Au_15_PEG, as it relates to proteostasis. Au_15_SG_13_ protected cells from the accumulation of protein aggregates, whereas Au_15_PEG increased aggregate formation. It should be noted that the surface modifications, physicochemical properties, shapes and hydrodynamic sizes differ profoundly for Au_15_SG_13_ and Au_15_PEG (Table S1). All of these parameters determine the nanoparticle interactions with cells and intracellular organelles 12. For example, PEGylation modulates the composition of the nanoparticle corona 59 and can enhance intracellular nanoparticle movement 60.

At present, the cellular mechanisms controlling the impact of Au_15_SG_13_ and Au_15_PEG on protein aggregation have not been identified. Since Au_15_SG_13_ and Au_15_PEG did not have opposing effects on lysosomal properties, we propose that AuNCs impact other pathways that also regulate proteostasis. Possible candidates are alternative routes of protein degradation, changes in protein synthesis, oxidative stress, and protein folding 61, 62. Future experiments will have to address these questions.

Gold nanoparticles can increase cellular ROS concentrations and stimulate the formation of protein aggregates 25, 63. Given that the incubation with 1 μM AuNCs for 24 hours diminished (Au_15_SG_13_) or increased (Au_15_PEG) protein aggregation (Figure 8A), we examined whether this could be explained by differences in ROS abundance. Indeed, when evaluated with two independent assays, we observed a slight ROS reduction for Au_15_SG_13_, but an increase for Au_15_PEG (Figure S9). This is consistent with the interpretation that AuNCs modulate protein aggregation at least in part through changes in cellular ROS concentrations.

### Effects of AuNCs on stress granule formation

The experiments described above support the idea that AuNCs can produce cellular stress which impinges on lysosomes, stress-activated transcription factors, and potentially protein aggregation. This prompted us to investigate additional branches of the integrated stress response. Nrf2 and protein aggregate formation are tightly linked to oxidative stress, and the increase in oxidative stress is frequently associated with the formation of cytoplasmic stress granules 26, 61. One of the pathways to clear stress granules involves autophagy, a process that depends on lysosomal function 64.

As described above, Au_15_SG_13_ altered lysosomal properties and diminished protein aggregation, both under normal conditions and in the presence of chloroquine. To define further the effects of Au_15_SG_13_ on oxidative stress responses, we examined whether Au_15_SG_13_ stimulates the assembly of cytoplasmic stress granules. Using HuR and importin-α1 as marker proteins, stress granule formation was not observed for different treatment times (Figure S10). The same results were obtained when U251N cells were exposed to Au_25_SG_18_.

Since Au_15_SG_13_ reduced protein aggregate formation induced by chloroquine (Figure 8B), we investigated whether these AuNCs also modulate the response to oxidative stress (Figure S11). To this end, sodium arsenite induced canonical stress granules in U251N cells pretreated with vehicle, Au_15_SG_13_ or Au_25_SG_18_ (Sodium arsenite rather than H_2_O_2_ was used, because H_2_O_2_ may fail to induce canonical stress granules 28).

Quantitative single-cell analyses revealed that 10 µM Au_15_SG_13_ and 10 µM Au_25_SG_18_ reduced slightly the abundance of importin-α1 in unstressed cells and in arsenite-induced stress granules. This effect was more pronounced for Au_25_SG_18_ than Au_15_SG_13_. Similarly, AuNCs marginally reduced the abundance of G3BP1, a stress granule nucleating protein 28, in nuclei and stress granules. Examination of individual stress granules did not uncover AuNC-dependent changes in granule size distribution (Figure S11C). Taken together, Au_15_SG_13_ and Au_25_SG_18_ did not provoke the formation of cytoplasmic stress granules. Moreover, both AuNCs had only minor impact on stress granule properties.

## Conclusions

AuNCs are promising nanostructures for biomedical applications and have been used to image glioblastoma and other tumors in rodents 32, 65, 66. Although AuNCs were reported to have low toxicity, in-depth studies to identify sub-lethal effects on cell physiology are sparse. We now show that AuNCs can modulate lysosomal parameters, specific aspects of the integrated stress response, and protein aggregation. Notably, these changes not only occur in the absence of overt cell killing, they are also -to a large extent- reversible. Some AuNCs are rapidly cleared by renal excretion 40 and could potentially damage the kidney. In human renal tubule cells, metabolic activities were somewhat reduced with Au_15_ SG_13,_ but Au_25_ SG_18_ caused no changes. Collectively, these findings indicate that the biological impact of AuNCs is determined by the number of gold atoms, size of the nanocluster, and properties of the surface modification. The results further emphasize possible cell type specific differences that are relevant to *in vivo* applications.

Our experiments focused on lysosomes and stress responses, because they are essential for overall cellular homeostasis and cell fate. Lysosomes are particularly relevant to tumor biology, as they are required to remove damaged proteins and organelles through autophagy. Autophagy in glioblastoma and other tumor cells can enhance tumor formation, whereas the inhibition of autophagy may trigger cancer cell death (reviewed in 67, 68). Accordingly, lysosomes have been targeted successfully to induce cancer cell death and overcome treatment resistance 68.

The current study is unique, as it conducts in-depth analyses of the cellular responses induced by AuNCs. In particular, we provide -for the first time- detailed information on how AuNCs modulate the properties of lysosomes. We uncovered homeostatic perturbations caused by AuNCs in glioblastoma cells and biological processes that are linked to lysosome performance. The internalization and the ensuing involvement of lysosomes represent the most plausible scenario for AuNCs. However, it should be noted that due to their ultra-small size the direct imaging of the single particles in cells has not been possible.

**Figure 9:**
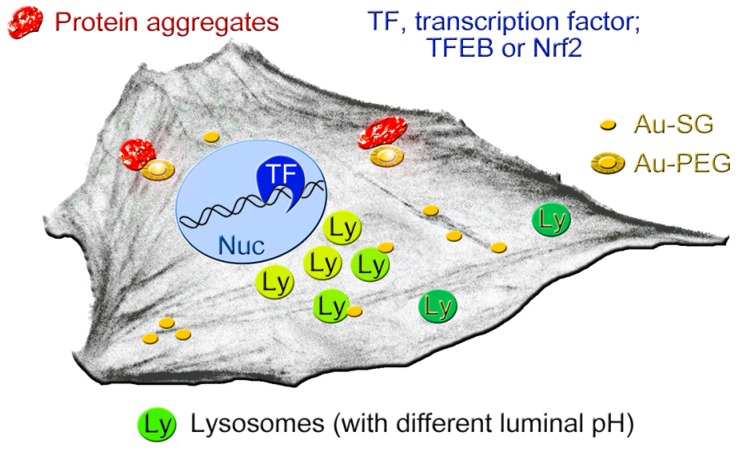
Simplified model depicting the cellular components that are affected, at least transiently, by AuNCs. AuNCs in this study were surface-modified with GSH (Au-SG) or PEGylated (Au-PEG). Lysosomes with more acidic pH (light green) are perinuclear, whereas peripheral lysosomes are less acidic (dark green). AuNCs stimulate the transient nuclear accumulation of transcription factors TFEB and Nrf2. PEGylated AuNCs may increase protein aggregation. See text for details.

The simplified model in Figure 9 depicts the cellular components that we identified as possible AuNC targets. These new insights provide a framework for further AuNC-based applications in nanooncology. This includes the safe use for the imaging of glioblastoma and other tumors. Our study also identified lysosomal homeostasis and proteostasis as potential targets for future AuNC-dependent cancer treatment.

## Methods

### Materials

Primary antibodies against the following antigens were purchased from the sources specified and used at the dilutions indicated: TFEB (Sigma-Aldrich, SAB4503154; diluted 1:500), LAMP1 (Abcam, ab24170; 1:1000). LAMP2 (Abcam, ab13524; 1:500), Nrf2 (Abcam, ab31163; 1:200), G3BP1 (BD Biosciences; 1:2000), HuR (Santa Cruz Biotechnology, sc-5261; 1:2000), importin-α1 (Santa Cruz Biotechnology, sc-6917; 1:500), α-tubulin (Santa Cruz Biotechnology, sc-5286; 1:500), STAT3 (Cell Signaling Technology, #9139; 1:1250). Secondary antibodies: AlexaFluor®647 anti-rabbit IgG (Life Technologies, A21244; 1:500) and AlexaFluor®647 anti-rat IgG (Life Technologies, A21247; 1:500), AlexaFluor®647 anti-mouse (Jackson ImmunoResearch, 715-605-150; 1:400), Cy3™-anti-rabbit (Jackson ImmunoResearch, 711-165-152; 1:250).

### Synthesis of AuNCs

Au_15_SG_13_ was synthesized as reported 69. Au_25_SG_18_ was synthesized as follows: 234 mg glutathione (GSH) was dissolved in 35ml methanol, 2 ml tributylamine and 2 ml triethylamine. Then 100mg HAuCl_4_•3H_2_O dissolved in 10ml of water was added. The solution was stirred 3 hours at 45°C and then solution cooled to room temperature. 50mg tetramethylammonium borohydride were added with vigorous stirring. After 1 hour, additional 25mg borohydride were added. The solution was stirred for 3 hours and then left overnight without agitation before purification. Precipitation of AuNCs was induced by adding 1ml of 10% NH_4_OH and diethyl ether. Unwanted products were removed through cycles of dissolution/precipitation/ centrifugation. The final precipitate was dissolved in a minimum of H_2_O/NH_4_OH and then precipitated with MeOH. After centrifugation, the powder was dissolved in 10 ml water. Then 2ml of glacial acetic acid was added, the solution was left undisturbed for 1 hour and then centrifuged (5 min at 10 000 rpm). The supernatant was collected and precipitated with MeOH. An additional cycle of dissolution/precipitation with H_2_O/NH_4_OH and acetic acid was performed and the powder dried under vacuum over P_2_O_5_.

### PEGylation of AuNCs

PEG_5000_-NH_2_ was grafted to the carboxylic acid of GSH by peptide coupling. Briefly, 25 mg AuNCs, 600 mg PEG_5000_-NH_2_ and 100 mg EDC•HCl (*N*-ethyl-*N*′-(3-(dimethylamino)propyl)carbodiimide hydrochloride) were dissolved in 5 ml water. The pH was adjusted to 7 with 1M NaOH, and the solution was stirred 24h at room temperature. Excess reagents were removed by dialysis, using a membrane with 10kDa cut-off (Sartorius).

### Cell culture

Human U251N GBM cells (American Type Culture Collection; Rockville, MD, USA) were cultured in Dulbecco's Modified Eagle Medium (DMEM; Life Technologies Inc. Burlington, ON, Canada) with the presence of 5% (v/v) fetal bovine serum (FBS; Gibco; Penrose, Auckland, New Zealand), supplemented with 1% penicillin-streptomycin (Pen/Strep, Gibco), unless indicated otherwise. Cells were incubated at 37^o^C with 5% CO_2_ and 95% relative humidity. For treatments with AuNCs or pharmacological agents control samples were incubated in the presence of the vehicle. “Vehicle” refers to the buffer or solvent used to disperse AuNCs or dissolve compounds. The volume of vehicle in the control was identical to the volume of dispersed AuNCs or dissolved agent. The incubation with vehicle, dispersed AuNCs or dissolved agents was always performed in growth medium.

### Evaluation of cell numbers and cell metabolic activity

Cells were seeded in 96-well cell plates (Costar, Corning, New York, USA) at a density of 5,000 cells/well in serum-supplemented medium (DMEM, 5% FBS, 1% Pen/Strep). After 24 hours, cells were treated for 72 hours with Au_15_SG_13_, Au_15_PEG, Au_25_SG_18_ and Au_25_PEG at different final concentrations (1 nM, 100 nM, 1 μM, 10 μM, 100 μM). Following 72-hour treatments, cells were fixed in 4% paraformaldehyde (PFA; BDH, Toronto, ON, Canada) for 10 minutes at room temperature. PFA was aspirated, and cells were stained with 10 μM Hoechst 33342 (Invitrogen, H1399, OR, USA) for 10 minutes at room temperature. Cells were washed with phosphate-buffered saline (PBS) and imaged with a Leica DMI 4000B fluorescence microscope (Leica microsystems, Heidelberg, Germany). Micrographs were analyzed with ImageJ 70.

Metabolic activities of U251N or HEK293 cells were measured with 3-(4,5-dimethylthiazol-2-yl)-2,5-diphenyltetrazolium bromide (MTT). Cells treated with vehicle or AuNCs were incubated for 1 hour at 37^o^C with 0.5 mg/mL MTT (M2128, Sigma-Aldrich), diluted in DMEM. Following incubation, medium was aspirated and replaced with dimethyl sulfoxide to solubilize the formazan product. Colorimetric measurements at 595 nm were performed with a Biochrom EZ Read 2000 Microplate Reader (Biochrom, Cambridge, United Kingdom).

### Fluorescence microscopy and immunocytochemistry

U251N cells were seeded at a density of 5,000 cells per 12 mm diameter coverslip. After 24 hours in 120 μL serum-supplemented DMEM (5% FBS, 1% Pen/Strep), medium was replaced with DMEM and cells were treated as indicated. Following treatment, medium was aspirated and cells were incubated with 10 μm Hoechst 33342 for nuclear staining and a second fluorescent dye to detect lysosomes, and cathepsin B activity. Following incubation with dyes, cells were washed twice with Earle's Balanced Salt Solution (EBSS) and imaged with a Leica fluorescence microscope. Micrographs were analyzed with ImageJ. Alternatively, images were acquired with a Zeiss LSM780 confocal microscope, and images were evaluated with MetaXpress® analysis software (Molecular Devices, San Jose, CA, USA) as published by us 71, 72. For immunocytochemistry, treated cells were fixed in 4% paraformaldehyde at room temperature and permeabilized with 0.1% Triton X-100 (Sigma-Aldrich) in PBS for 10 minutes. Cells were blocked for 1 hour in 10% goat serum (Gibco). Incubation with primary antibodies was overnight at 4°C for 24 hours. Cells were washed three times for 5 minutes with PBS and incubated with secondary antibodies for 1 hour at room temperature. Nuclei were detected with 10 μM Hoechst 33342 and F-actin with 1:50 Alexa Fluor® 488 Phalloidin (Invitrogen). Coverslips were mounted in Aqua-PolyMount (PolySciences, Warrington, PA, USA) or Vectashield® (Vector Laboratories, Burlingame, CA, USA).

The detection of STAT3 was essentially as described 46; all steps were carried out at room temperature. In brief, upon incubation with vehicle, AuNCs or chloroquine, cells were rinsed twice with PBS and fixed for 15 min in 4% paraformaldehyde. Fixed cells were permeabilized 5 minutes with 0.1% saponin (Sigma-Aldrich) in PBS containing 2 mg/ml BSA and 1 mM NaN_3_. Samples were blocked 1 hour in PBS/5% FBS/1 mM NaN_3_ (blocking buffer). All subsequent steps were carried out in blocking buffer.

### Staining of lysosomes with LysoTracker DND-99

Following treatment, growth medium was aspirated and cells were incubated with 10 μm Hoechst 33342 and labeled with 50 nM LysoTracker® Red DND-99 (Invitrogen, Eugene, Oregon, USA) for 20 minutes at 37^o^C.

### Evaluation of acidic organelles

LysoSensor™ DND-189 (Invitrogen) has a pK_a_ of 5.2; the compound is fluorescent when located in acidic vesicles, such as lysosomes. Following treatment, growth medium was aspirated, and cells were incubated with 10 μm Hoechst 33342 and labeled with 1 μM LysoSensor™ DND-189 for 20 minutes at 37^o^C.

### Analysis of lysosome subcellular distribution

Nuclear boundaries were determined with Hoechst 33342. The perinuclear area was demarcated with ImageJ. It is the region within a 5 µm distance from the nuclear margin; the cellular region outside of this zone is defined as the peripheral area 53. Lysosome distribution was determined as the LysoTracker® DND-99 fluorescence in the perinuclear/LysoTracker® DND-99 fluorescence in the peripheral area.

### Measurement of cytosolic pH in living cells

The cytosolic pH was determined with SNARF®-1 (5-(and-6)-Carboxy SNARF™-1, acetoxymethyl ester, acetate; C1272, ThermoFisher), which is suitable for ratiometric pH measurements. SNARF®-1 was excited at 488 nm and emissions were recorded at 580 nm and 640 nm, essentially as described 73. For calibration, U251N cells were seeded in 96-well plates, containing 10,000 cells/well. After overnight growth, samples were incubated with 5 μM SNARF®-1 in serum-free/phenol-free DMEM (45 min, 37^o^C). Calibration was performed for different pH values (pH 5.5, 6.0, 6.5, 7.0, 7.1, 7.2, 7.3, 7.4, 7.5) in the presence of 10 μM nigericin (Sigma) 73. Fluorescence intensities were measured with a SPARK10M microplate reader (excitation: 485 nm, bandwidth 20 nm; emission E_1_: 635 nm, bandwidth 35 nm; and emission E_2_: 580 nm, bandwidth 20 nm). The ratio E_1_/E_2_ for fluorescence emission was plotted as a function of pH, and non-linear regression was used for curve-fitting. The cytosolic pH values of U251N cells were extrapolated from the calibration curve.

### Measurement of cathepsin B activity

Dequenching of the fluorogenic substrate MR-(RR)_2_ (Magic Red, ImmunoChemistry Technologies; Bloomington, MN, USA) determined the activity of the lysosomal protease cathepsin B. Following treatment, growth medium was aspirated, and cells were incubated with 10 μm Hoechst 33342 and Magic Red (1:260) for 30 minutes at 37^o^C.

### Proteostat assay

The assay followed the manufacturer's protocol with minor modifications. Specifically, cells were incubated with the Proteostat® reagent (1:8000; ENZ51035; Enzo Life Sciences; Farmingdale, NY, USA) for 2 hours at room temperature. Nuclei were detected with 10 μM Hoechst 33342, and F-actin with 1:50 Alexa Fluor® 488 Phalloidin (Invitrogen).

### Measurement of cellular ROS with CellRox® Green and CM-H_2_DCFDA

U251N cells were incubated with 1 μM Au_15_SG_13_ or Au_15_PEG for 24 hours in serum-free DMEM at 37^o^C; 2.5 µM CellRox® Green (ThermoFisher) was added during the last 30 minutes of the incubation period. Microscopic images were acquired and pixel intensities were quantified per nuclear area as described by us 74.

ROS were also detected with CM-H_2_DCFDA (ThermoFisher). U251N cells were incubated with 1 μM CM-H_2_DCFDA in serum-free/phenol-free DMEM for 30 min at 37^o^C, washed once with PBS and treated with 1 μM Au_15_SG_13_ or Au_15_PEG for 24 hours in serum-free/phenol-free DMEM. Cells were washed once with PBS and imaged live with a Leica microscope at 63X objective.

### Measurements of cell size and stress granule parameters

Oxidative stress was induced by incubating U251N cells for 2 hours with growth medium containing 0.5 mM sodium arsenite. Control cells received water instead of sodium arsenite. Cell size measurements and the detection of cytoplasmic stress granules followed our published protocols 71, 72. The measurements were performed for 112 to149 cells and 1448 to 2422 stress granules for each condition.

### Statistical analysis

Data are shown as average ± standard error of the mean (SEM). Student's t-test or One-Way ANOVA with Bonferroni correction was used to identify significant differences. A *p* value <0.05 was considered statistically significant. Significant changes are indicated in the figures as follows: *p<0.05, **p<0.01, ***p<0.001.

## Supplementary Material

Supplementary figures and table.

## References

[1] Nandi S, Ghosh S, Bhattacharyya K (2018). Live Cell Microscopy: A Physical Chemistry Approach. J Phys Chem B.

[2] Vankayala R, Kuo C-L, Nuthalapati K, Chiang C-S, Hwang KC (2015). Nucleus-Targeting Gold Nanoclusters for Simultaneous In Vivo Fluorescence Imaging, Gene Delivery, and NIR-Light Activated Photodynamic Therapy. Adv Funct Mater.

[3] Jin R, Zeng C, Zhou M, Chen Y (2016). Atomically Precise Colloidal Metal Nanoclusters and Nanoparticles: Fundamentals and Opportunities. Chem Rev.

[4] Su Y, Xue T, Liu Y, Qi J, Jin R, Lin Z (2019). Luminescent metal nanoclusters for biomedical applications. Nano Research.

[5] Zheng Y, Lai L, Liu W, Jiang H, Wang X (2017). Recent advances in biomedical applications of fluorescent gold nanoclusters. Adv Colloid Interface Sci.

[6] Kang X, Chong H, Zhu M (2018). Au25(SR)18: the captain of the great nanocluster ship. Nanoscale.

[7] Crawford SE, Hartmann MJ, Millstone JE (2019). Surface Chemistry-Mediated Near-Infrared Emission of Small Coinage Metal Nanoparticles. Acc Chem Res.

[8] Kang X, Zhu M (2019). Tailoring the photoluminescence of atomically precise nanoclusters. Chem Soc Rev.

[9] Liu CP, Wu TH, Lin YL, Liu CY, Wang S, Lin SY (2016). Tailoring Enzyme-Like Activities of Gold Nanoclusters by Polymeric Tertiary Amines for Protecting Neurons Against Oxidative Stress. Small.

[10] Ao H, Feng H, Zhao M, Zhao M, Chen J, Qian Z (2017). Redox-Triggered Bonding-Induced Emission of Thiol-Functionalized Gold Nanoclusters for Luminescence Turn-On Detection of Molecular Oxygen. ACS Sens.

[11] Zeng C, Chen Y, Das A, Jin R (2015). Transformation Chemistry of Gold Nanoclusters: From One Stable Size to Another. J Phys Chem Lett.

[12] Kodiha M, Wang YM, Hutter E, Maysinger D, Stochaj U (2015). Off to the organelles - killing cancer cells with targeted gold nanoparticles. Theranostics.

[13] Wang J, Zhang G, Li Q, Jiang H, Liu C, Amatore C (2013). In vivo self-bio-imaging of tumors through in situ biosynthesized fluorescent gold nanoclusters. Sci Rep.

[14] Lawrence RE, Zoncu R (2019). The lysosome as a cellular centre for signalling, metabolism and quality control. Nat Cell Biol.

[15] Oyarzun JE, Lagos J, Vazquez MC, Valls C, De la Fuente C, Yuseff MI (2019). Lysosome motility and distribution: Relevance in health and disease. Biochim Biophys Acta Mol Basis Dis.

[16] Xu H, Ren D (2015). Lysosomal physiology. Annu Rev Physiol.

[17] Raben N, Puertollano R (2016). TFEB and TFE3: Linking Lysosomes to Cellular Adaptation to Stress. Annu Rev Cell Dev Biol.

[18] Perera RM, Zoncu R (2016). The Lysosome as a Regulatory Hub. Annu Rev Cell Dev Biol.

[19] Samie MA, Xu H (2014). Lysosomal exocytosis and lipid storage disorders. J Lipid Res.

[20] Settembre C, Ballabio A (2014). Lysosome: regulator of lipid degradation pathways. Trends Cell Biol.

[21] Bonifacino JS, Neefjes J (2017). Moving and positioning the endolysosomal system. Curr Opin Cell Biol.

[22] Cabukusta B, Neefjes J (2018). Mechanisms of lysosomal positioning and movement. Traffic.

[23] Umair M, Javed I, Rehman M, Madni A, Javeed A, Ghafoor A (2016). Nanotoxicity of Inert Materials: The Case of Gold, Silver and Iron. J Pharm Pharm Sci.

[24] Kodiha M, Mahboubi H, Maysinger D, Stochaj U (2016). Gold Nanoparticles Impinge on Nucleoli and the Stress Response in MCF7 Breast Cancer Cells. Nanobiomedicine.

[25] Samhadaneh DM, Alqarni KA, Smart A, Kuang M, Moujaber O, Maysinger D (2019). Gold nanourchins induce cellular stress, impair proteostasis and damage RNA. Nanomed Nanotechnol Biol Med.

[26] Schmidlin CJ, Dodson MB, Madhavan L, Zhang DD (2019). Redox regulation by NRF2 in aging and disease. Free Radic Biol Med.

[27] Traboulsi H, Guerrina N, Iu M, Maysinger D, Ariya P, Baglole CJ (2017). Inhaled Pollutants: The Molecular Scene behind Respiratory and Systemic Diseases Associated with Ultrafine Particulate Matter. Int J Mol Sci.

[28] Mahboubi H, Stochaj U (2017). Cytoplasmic stress granules: Dynamic modulators of cell signaling and disease. Biochim Biophys Acta.

[29] Protter DSW, Parker R (2016). Principles and Properties of Stress Granules. Trends Cell Biol.

[30] Anderson P, Kedersha N, Ivanov P (2015). Stress granules, P-bodies and cancer. Biochim Biophys Acta.

[31] Cloer EW, Goldfarb D, Schrank TP, Weissman BE, Major MB (2019). NRF2 Activation in Cancer: From DNA to Protein. Cancer Res.

[32] Cifuentes-Rius A, Ivask A, Das S, Penya-Auladell N, Fabregas L, Fletcher NL (2017). Gold Nanocluster-Mediated Cellular Death under Electromagnetic Radiation. ACS Appl Mater Interfaces.

[33] Pyo K, Thanthirige VD, Kwak K, Pandurangan P, Ramakrishna G, Lee D (2015). Ultrabright Luminescence from Gold Nanoclusters: Rigidifying the Au(I)-Thiolate Shell. Journal of the American Chemical Society.

[34] Bertorelle F, Moulin C, Soleilhac A, Comby-Zerbino C, Dugourd P, Russier-Antoine I (2018). Bulky Counterions: Enhancing the Two-Photon Excited Fluorescence of Gold Nanoclusters. ChemPhysChem.

[35] Soleilhac A, Bertorelle F, Antoine R (2018). Sizing protein-templated gold nanoclusters by time resolved fluorescence anisotropy decay measurements. Spectrochim Acta A Mol Biomol Spectrosc.

[36] Soleilhac A, Bertorelle F, Dugourd P, Girod M, Antoine R (2017). Monitoring methanol-induced protein unfolding by fluorescence anisotropy measurements of covalently labelled rhodamine probe. Eur Phys J D.

[37] Soleilhac A, Bertorelle F, Comby-Zerbino C, Chirot F, Calin N, Dugourd P (2017). Size Characterization of Glutathione-Protected Gold Nanoclusters in the Solid, Liquid and Gas Phases. J Phys Chem C.

[38] Kang X, Zhu M (2019). Tailoring the photoluminescence of atomically precise nanoclusters. Chem Soc Rev.

[39] Yu M, Xu J, Zheng J (2019). Renal Clearable Luminescent Gold Nanoparticles: From the Bench to the Clinic. Angew Chem Int Ed Engl.

[40] Du B, Jiang X, Das A, Zhou Q, Yu M, Jin R (2017). Glomerular barrier behaves as an atomically precise bandpass filter in a sub-nanometre regime. Nat Nanotechnol.

[41] Ji J, Moquin A, Bertorelle F, Ky Chang P, Antoine R, Luo J (2019). Organotypic and primary neural cultures as models to assess effects of different gold nanostructures on glia and neurons. Nanotoxicology.

[42] Eskelinen EL, Tanaka Y, Saftig P (2003). At the acidic edge: emerging functions for lysosomal membrane proteins. Trends Cell Biol.

[43] Pierzynska-Mach A, Janowski PA, Dobrucki JW (2014). Evaluation of acridine orange, LysoTracker Red, and quinacrine as fluorescent probes for long-term tracking of acidic vesicles. Cytometry A.

[44] Johnson DE, Ostrowski P, Jaumouille V, Grinstein S (2016). The position of lysosomes within the cell determines their luminal pH. J Cell Biol.

[45] Lin HJ, Herman P, Kang JS, Lakowicz JR (2001). Fluorescence lifetime characterization of novel low-pH probes. Anal Biochem.

[46] Liu B, Palmfeldt J, Lin L, Colaco A, Clemmensen KKB, Huang J (2018). STAT3 associates with vacuolar H(+)-ATPase and regulates cytosolic and lysosomal pH. Cell Res.

[47] Chatr-Aryamontri A, Breitkreutz BJ, Oughtred R, Boucher L, Heinicke S, Chen D (2015). The BioGRID interaction database: 2015 update. Nucleic Acids Res.

[48] Ba Q, Raghavan G, Kiselyov K, Yang G (2018). Whole-Cell Scale Dynamic Organization of Lysosomes Revealed by Spatial Statistical Analysis. Cell Rep.

[49] Mohapatra L, Goode BL, Jelenkovic P, Phillips R, Kondev J (2016). Design Principles of Length Control of Cytoskeletal Structures. Annu Rev Biophys.

[50] Rivero F, Koppel B, Peracino B, Bozzaro S, Siegert F, Weijer CJ (1996). The role of the cortical cytoskeleton: F-actin crosslinking proteins protect against osmotic stress, ensure cell size, cell shape and motility, and contribute to phagocytosis and development. J Cell Sci.

[51] Cantwell H, Nurse P (2019). A systematic genetic screen identifies essential factors involved in nuclear size control. PLoS Genet.

[52] Palmieri M, Impey S, Kang H, di Ronza A, Pelz C, Sardiello M (2011). Characterization of the CLEAR network reveals an integrated control of cellular clearance pathways. Hum Mol Genet.

[53] Willett R, Martina JA, Zewe JP, Wills R, Hammond GRV, Puertollano R (2017). TFEB regulates lysosomal positioning by modulating TMEM55B expression and JIP4 recruitment to lysosomes. Nat Commun.

[54] Vega-Rubin-de-Celis S, Pena-Llopis S, Konda M, Brugarolas J (2017). Multistep regulation of TFEB by MTORC1. Autophagy.

[55] Kim S, Choi KJ, Cho SJ, Yun SM, Jeon JP, Koh YH (2016). Fisetin stimulates autophagic degradation of phosphorylated tau via the activation of TFEB and Nrf2 transcription factors. Sci Rep.

[56] Covas G, Marinho HS, Cyrne L, Antunes F (2013). Activation of Nrf2 by H2O2: de novo synthesis versus nuclear translocation. Methods Enzymol.

[57] Dong L, Li M, Zhang S, Li J, Shen G, Tu Y (2015). Cytotoxicity of BSA-Stabilized Gold Nanoclusters: In Vitro and In Vivo Study. Small.

[58] Levy JMM, Towers CG, Thorburn A (2017). Targeting autophagy in cancer. Nat Rev Cancer.

[59] Partikel K, Korte R, Stein NC, Mulac D, Herrmann FC, Humpf HU (2019). Effect of nanoparticle size and PEGylation on the protein corona of PLGA nanoparticles. Eur J Pharm Biopharm.

[60] Suh J, Choy KL, Lai SK, Suk JS, Tang BC, Prabhu S (2007). PEGylation of nanoparticles improves their cytoplasmic transport. Int J Nanomedicine.

[61] Reichmann D, Voth W, Jakob U (2018). Maintaining a Healthy Proteome during Oxidative Stress. Mol Cell.

[62] Klaips CL, Jayaraj GG, Hartl FU (2018). Pathways of cellular proteostasis in aging and disease. J Cell Biol.

[63] Lopez-Chaves C, Soto-Alvaredo J, Montes-Bayon M, Bettmer J, Llopis J, Sanchez-Gonzalez C (2018). Gold nanoparticles: Distribution, bioaccumulation and toxicity. In vitro and in vivo studies. Nanomedicine.

[64] Alberti S, Mateju D, Mediani L, Carra S (2017). Granulostasis: Protein Quality Control of RNP Granules. Front Mol Neurosci.

[65] Hu H, Huang P, Weiss OJ, Yan X, Yue X, Zhang MG (2014). PET and NIR optical imaging using self-illuminating (64)Cu-doped chelator-free gold nanoclusters. Biomaterials.

[66] Le Guevel X, Henry M, Motto-Ros V, Longo E, Montanez MI, Pelascini F (2018). Elemental and optical imaging evaluation of zwitterionic gold nanoclusters in glioblastoma mouse models. Nanoscale.

[67] Lahiri V, Hawkins WD, Klionsky DJ (2019). Watch What You (Self-) Eat: Autophagic Mechanisms that Modulate Metabolism. Cell Metab.

[68] Cordani M, Somoza A (2019). Targeting autophagy using metallic nanoparticles: a promising strategy for cancer treatment. Cell Mol Life Sci.

[69] Russier-Antoine I, Bertorelle F, Vojkovic M, Rayane D, Salmon E, Jonin C (2014). Non-linear optical properties of gold quantum clusters. The smaller the better. Nanoscale.

[70] Schindelin J, Arganda-Carreras I, Frise E, Kaynig V, Longair M, Pietzsch T (2012). Fiji: an open-source platform for biological-image analysis. Nat Methods.

[71] Mahboubi H, Kodiha M, Stochaj U (2013). Automated detection and quantification of granular cell compartments. Microsc Microanal.

[72] Moujaber O, Fishbein F, Omran N, Liang Y, Colmegna I, Presley JF (2019). Cellular senescence is associated with reorganization of the microtubule cytoskeleton. Cell Mol Life Sci.

[73] Lucien F, Harper K, Pelletier PP, Volkov L, Dubois CM (2014). Simultaneous pH measurement in endocytic and cytosolic compartments in living cells using confocal microscopy.

[74] Kodiha M, Bednarz K, Maysinger D, Stochaj U (2017). Microscopy and Quantitative Imaging: Novel Applications in Health Research. Mendez-Vilas A, editor.

[75] Soleilhac A, Bertorelle F, Antoine R (2018). Sizing protein-templated gold nanoclusters by time resolved fluorescence anisotropy decay measurements. Spectrochim Acta A Mol Biomol Spectrosc.

