# Supplementary material for: Gold nanoclusters elicit homeostatic perturbations in glioblastoma cells and adaptive changes of lysosomes
Source: Theranostics. 2020 Jan 1;10(4):1633–48. doi: 10.7150/thno.37674 (PMC6993243; doi:10.7150/thno.37674)
Supplement: Supplementary file 1 — Supplementary figures and table. [file thnov10p1633s1.pdf]

Supplementary Information

Gold nanoclusters elicit homeostatic perturbations in glioblastoma cells and adaptive changes of lysosomes

Dusica Maysinger[1#*], Evan R. Gran[1#], Franck Bertorelle[2], Hussein Fakhouri[2], Rodolphe Antoine[2], Esha S. Kaul[1], Dana M. Samhadaneh[3], and Ursula Stochaj[3*]

McGill University, Pharmacology & Therapeutics, Montreal, Canada[1]

Université Lyon, CNRS, Institut Lumière Matière, Lyon, France[2]

McGill University, Physiology, Montreal, Canada[3]

[#]Both authors contributed equally.

*Corresponding authors.

Table S1. Hydrodynamic size measurements and zeta potential for Au_{15}NCs and Au_{25}NCs.

Hydrodynamic diameters were determined with time-resolved fluorescence anisotropy, according to our published protocols [75]. The zeta potential was measured with a Malvern Zetasizer Nano ZS; it was negative for all AuNCs studied here.

AuNC	Hydrodynamic diameter [nm]	Shape	Zeta [mV]
$Au_{15}SG_{13}$	2.9	asymmetric	-3.86
$Au_{15}SG_{13}$-nPEO_{5000} (Au_{15}PEG)	6	spherical	-2.14
$Au_{25}SG_{18}$	3.3	spherical	-7.46
$Au_{25}SG_{18}$-nPEO_{5000} (Au_{25}PEG)	4.5	spherical	-15.4

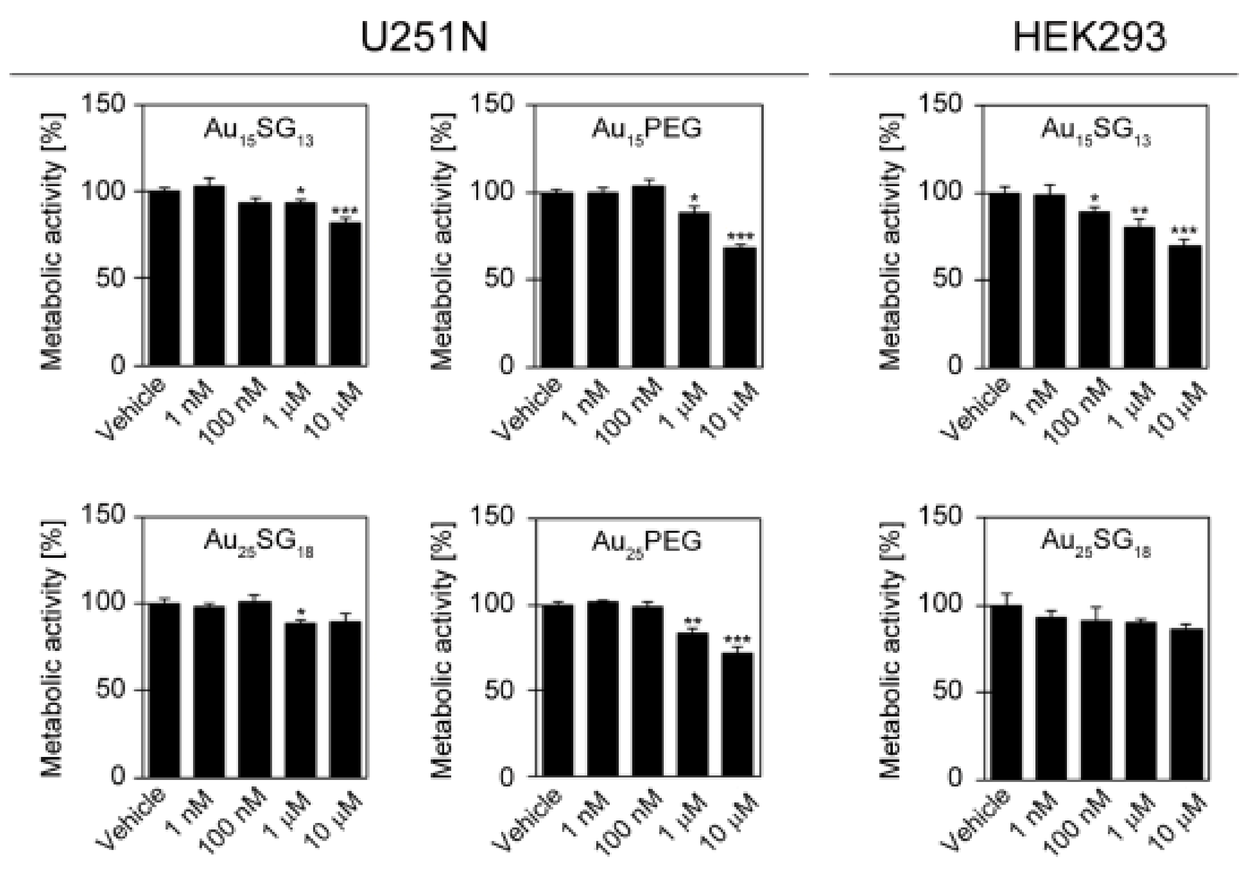

Figure S1. Effect of AuNCs on the metabolic activity of U251N and HEK293 cells.

U251N cells incubated for 72 hours with AuNCs at the final concentrations indicated, using the methods described for Figure 2. The MTT assay assessed metabolic activities for at least two independent experiments, with triplicate samples for each data set. Results are shown as average + SEM; *, p<0.05; **, p<0.01; ***, p<0.001.

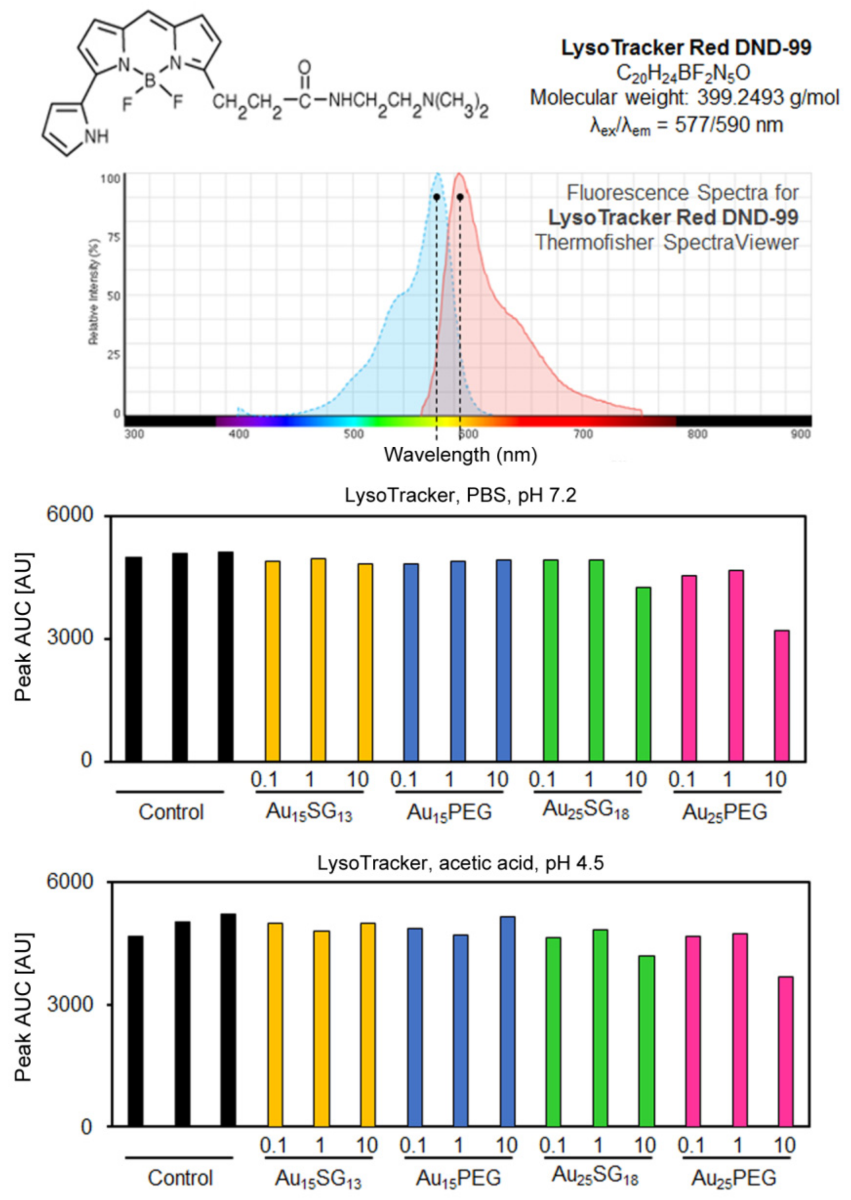

Figure S2. Effect of AuNCs on LysoTracker® Red DND-99 fluorescence.

The fluorescence intensity of 100 nM LysoTracker Red in PBS (pH 7.2) or 100 mM sodium acetate buffer (pH 4.5) was measured in the presence of different AuNC concentrations as indicated. Excitation was at 576 nm, emission spectra were measured with a Cary Eclipse Fluorescence Spectrophotometer (Agilent Technologies) between 586 to 700 nm using an excitation slit of 10 nm and an emission slit of 10 nm with a photomultiplier tube voltage of 600 V. The area under the curve (AUC) was obtained by integrating the emission spectra between 586 and 700 nm. AuNCs did not quench LysoTracker Red DND-99 fluorescence at concentrations up to 10 µM. AU; arbitrary units.

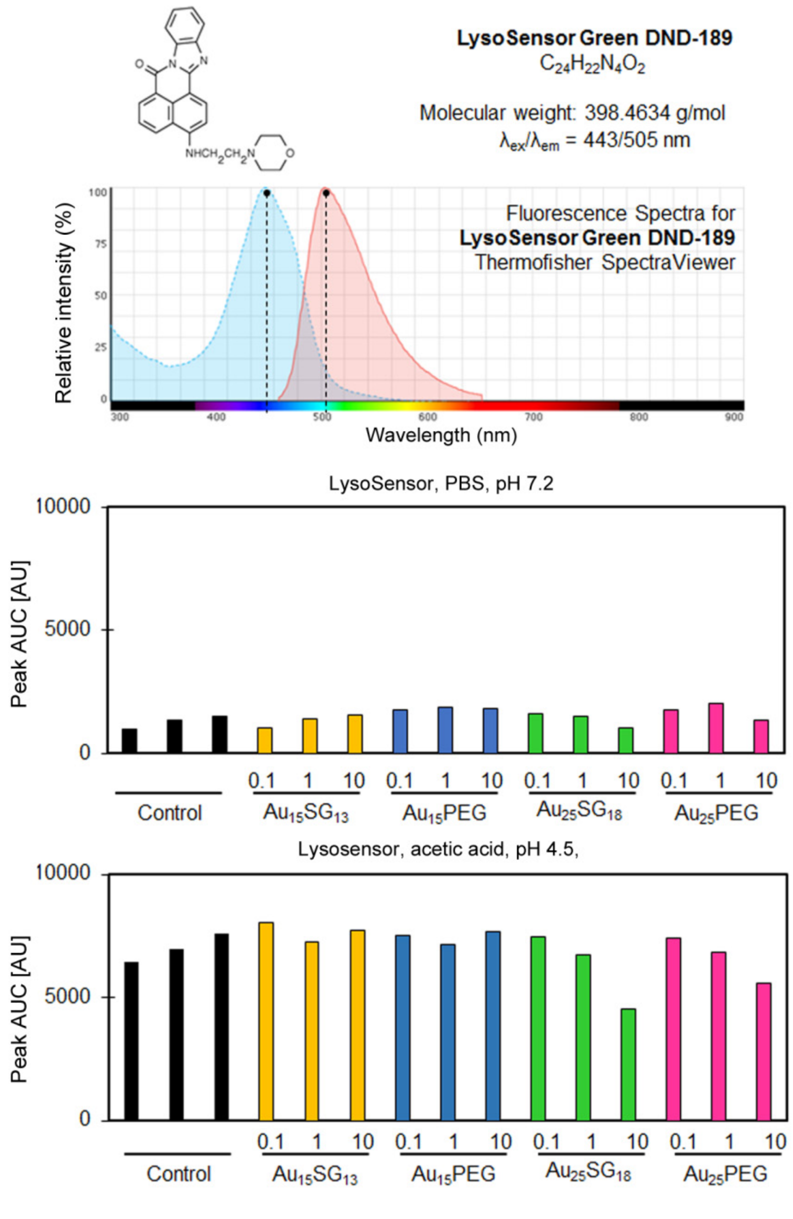

Figure S3. Effect of AuNCs on LysoSensor™ Green DND-189 fluorescence.

The fluorescence intensity of 1 µM LysoSensor green in PBS (pH 7.2) or 100 mM acetate buffer (pH 4.5) was measured with a Cary Eclipse Fluorescence Spectrophotometer (Agilent Technologies) in the presence of different AuNC concentrations as indicated. Excitation was at 455 nm; emission was measured between 460 and 650 nm using excitation slit of 5 nm and an emission slit of 5 nm with a photomultiplier tube voltage of 600 V. The area under the curve (AUC) was obtained by integrating the emission spectra between 460 and 650 nm. AuNCs did not quench LysoSensor fluorescence at AuNC concentrations up to 10 µM. AU; arbitrary units.

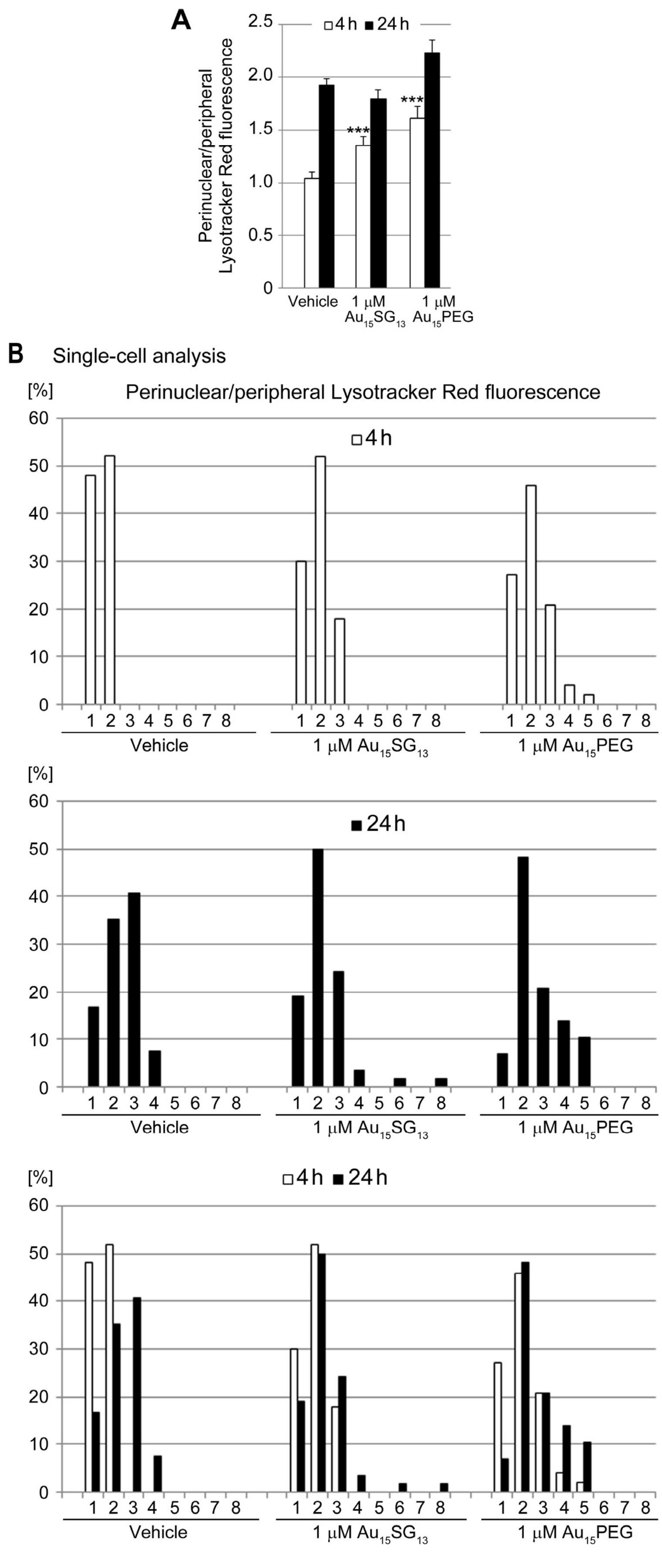

Figure S4. Detailed analysis of Lysotracker Red signal distribution.

An in-depth evaluation was performed for the perinuclear and peripheral localization of lysosomes. The assessment was carried out on raw data calculated for the perinuclear/ peripheral ratio of Lysotracker Red signals.

(A) Bar graph depicting the ratio for vehicle, 1 µM $Au_{15}SG_{13}$ or 1 µM $Au_{15}PEG$ at 4 and 24 hours. Bars represent average ± SEM; in two independent experiments 43 to 58 cells were assessed for each condition; ***p<0.001.

(B) The perinuclear/peripheral ratio of Lysotracker Red was evaluated for single cells and results of two independent experiments were binned. All bins have equal size. For example, bin 1 represents cells with a ratio of 0 to <1; bin 2 contains cells with a ratio of 1 to <2. The bars depict the percentage of cells in each bin.

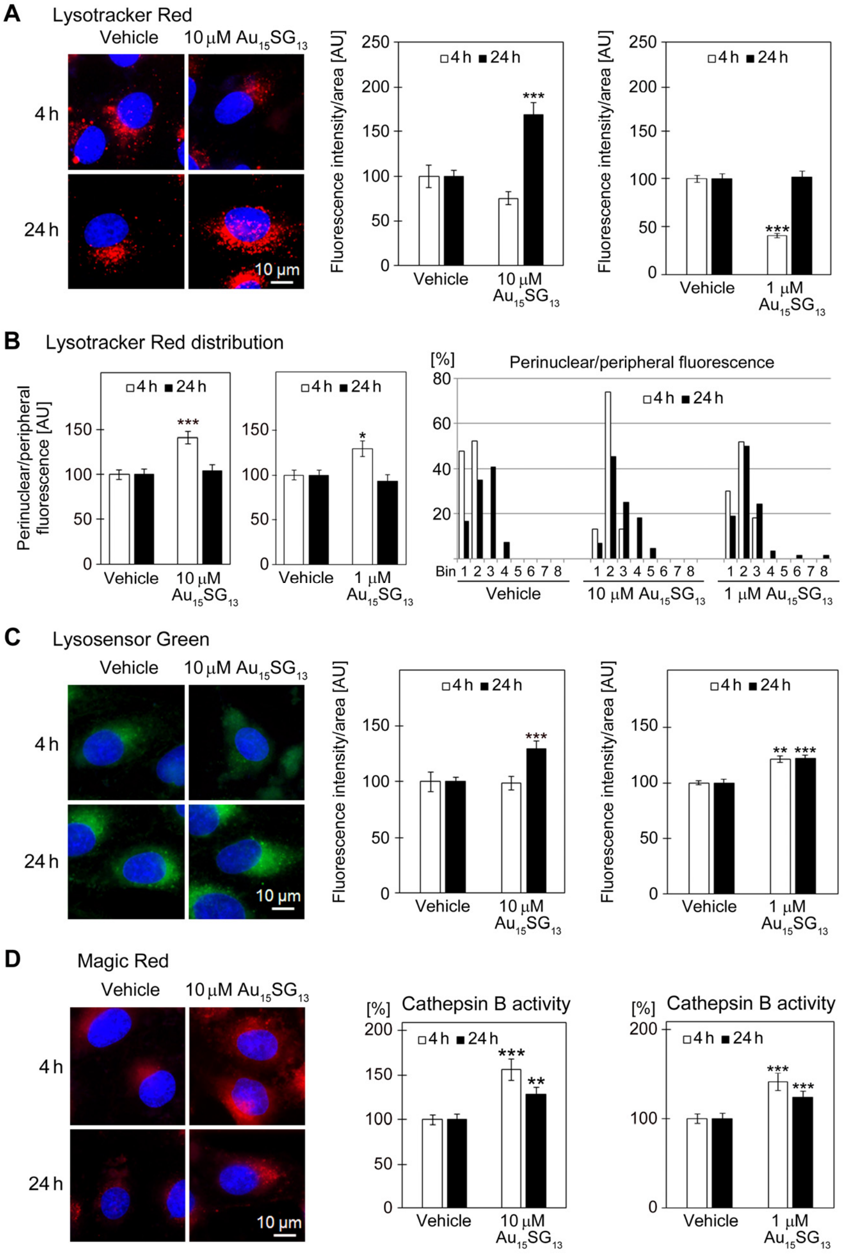

Figure S5. Impact of 10 μM $Au_{15}SG_{13}$ on the properties of acidic vesicles.

U251N cells were treated and analyzed as described for Figures 3 and 6, with a final concentration of 10 μM $Au_{15}SG_{13}$. For comparison, bar graphs with the results for 1 μM $Au_{15}SG_{13}$ are also shown (see main manuscript). (A) Between 22 and 49 cells were scored with Lysotracker Red for each data point in two independent experiments. (B) Lysotracker Red distribution was determined for at least 38 cells per condition and two independent experiments. Binning was performed as described for Figure S4. (C) Lysosensor Green signals were quantified for 24 to 41 cells for each condition. One representative experiment was evaluated for the 4-hour time point; two independent experiments were assessed for the 24-hour incubation period. (D) Cathepsin B activity was quantified for at 53 to 92 cells per condition. Results for two independent experiments are depicted as average ± SEM; *$p<0.05$, **$p<0.01$, ***$p<0.001$.

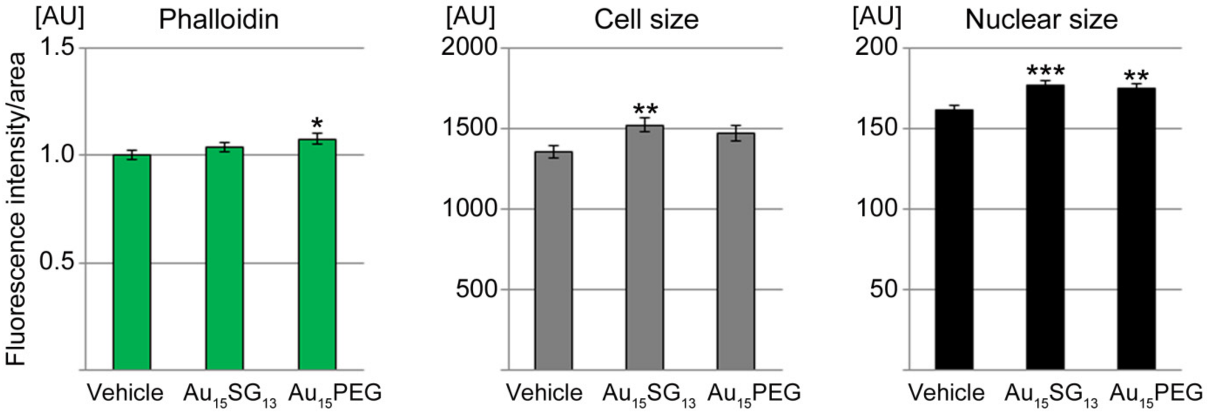

Figure S6. Effects of 1 µM $Au_{15}SG_{13}$ and $Au_{15}PEG$ on F-actin formation, cell and nuclear size.

U251N cells were incubated 24 hours with vehicle, 1 µM $Au_{15}SG_{13}$ or 1 µM $Au_{15}PEG$. Cells were fixed, processed and imaged as described for Figure 5. F-actin was stained with Alexa Fluor® 488 phalloidin.

Fluorescence intensities/cell area, cell and nuclear size were quantified. Results are shown for a representative experiment as average ± SEM. Between 97 and 103 cells were assessed per condition for phalloidin fluorescence and cell size. The size of nuclei was measured for 170 to 194 cells for each data point. Student's t-test uncovered significant differences between the vehicle control and AuNC-treated cells; *, $p<0.05$; **, $p<0.01$; **, $p<0.001$. AU, arbitrary units.

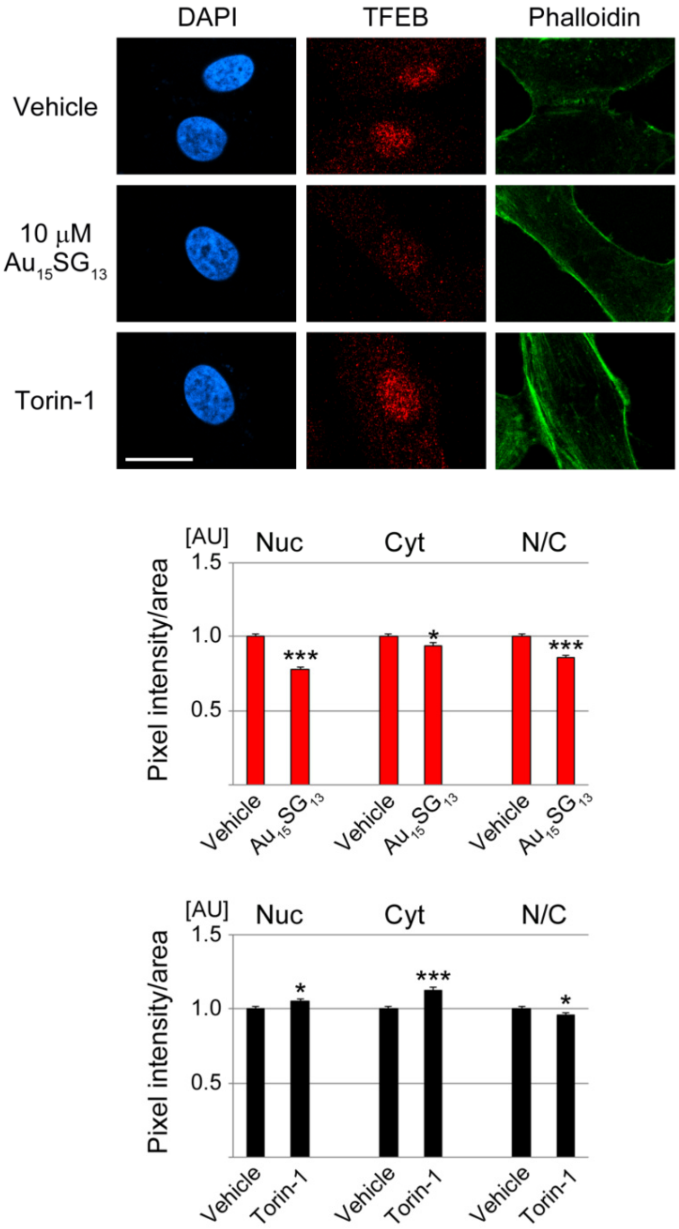

Figure S7. Effects of $Au_{15}SG_{13}$ and the mTOR inhibitor Torin-1 on the nucleocytoplasmic distribution of TFEB.

Immunocytochemistry located TFEB in U251N cells treated for 24 hours with vehicle (175 cells), 10 µM $Au_{15}SG_{13}$ (205 cells) or 2 µM Torin-1 (165 cells). DAPI stained the nucleus; scale bar is 20 µm. Pixel intensities in the nucleus/area (Nuc), cytoplasm/area (Cyt) and the ratio nuclear/cytoplasmic fluorescence (N/C) was determined for one representative experiment. Results normalized to the vehicle control are depicted as average ± SEM. Student's t-test identified significant differences between vehicle and AuNC-treated samples; *, $p<0.05$; ***, $p<0.001$. AU, arbitrary units.

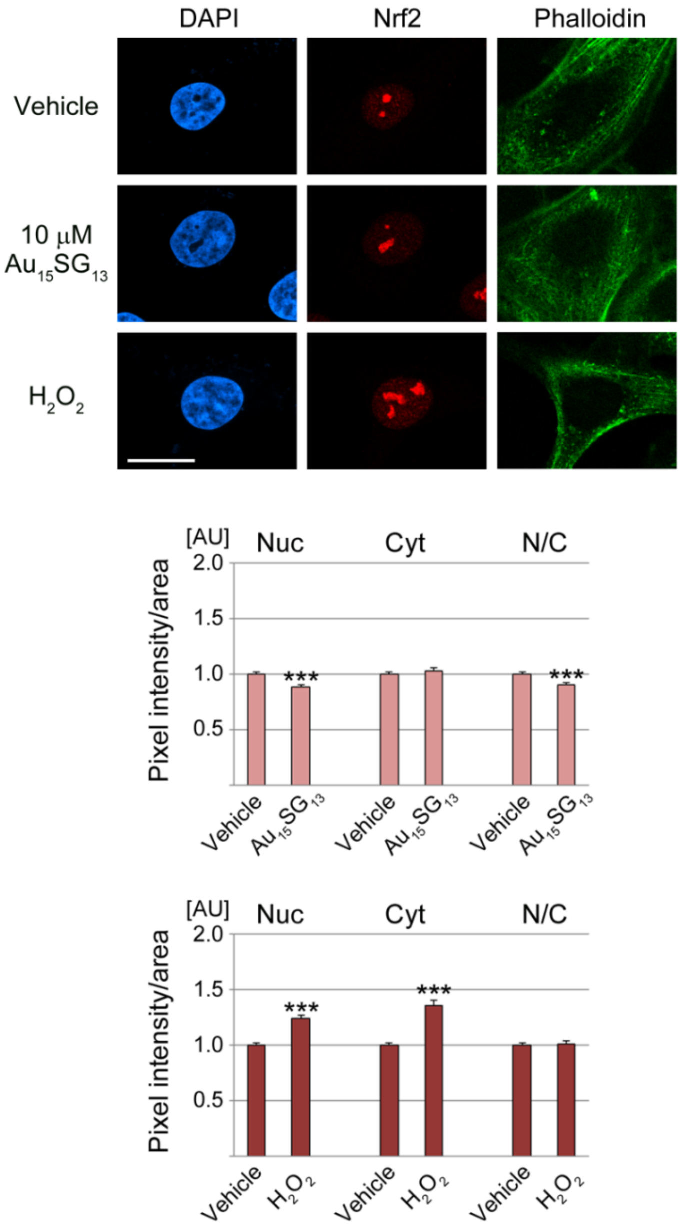

Figure S8. Effects of $Au_{15}SG_{13}$ and hydrogen peroxide on the nucleocytoplasmic distribution of Nrf2.

Immunocytochemistry located Nrf2 in U251N cells treated for 24 hours with vehicle (196 cells), 10 μM $Au_{15}SG_{13}$ (179 cells) or 300 μM hydrogen peroxide (165 cells). DAPI demarcated nuclei; scale bar is 20 μm. Pixel intensities in the nucleus/area (Nuc), cytoplasm/area (Cyt) and the ratios of nuclear/cytoplasmic fluorescence (N/C) were determined for one representative experiment. Results normalized to the vehicle control are shown as average ± SEM. Student's t-test identified significant differences between vehicle and AuNC-treated samples; ***, p<0.001.

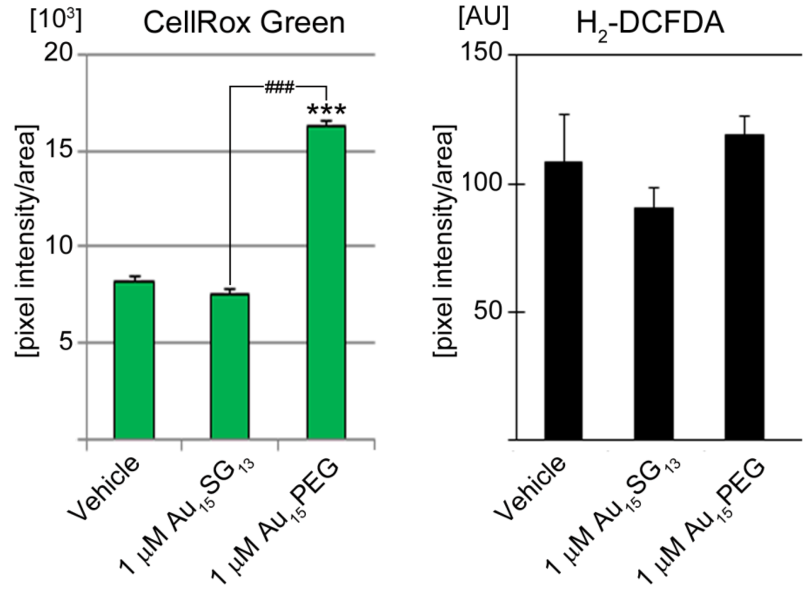

Figure S9. AuNC-dependent production of reactive oxygen species (ROS).

U251N cells were treated with vehicle, 1 µM $Au_{15}SG_{13}$ or 1 µM $Au_{15}PEG$ for 24 hours, as in Figure 8. CellRox® Green or H_2-DCFDA was used to monitor changes in ROS abundance. Microscopic images were acquired and pixel intensities were quantified per nuclear area (CellRox® Green) or per cell area (H_2-DCFDA). ROS production was evaluated for a typical experiment with CellRox® Green (192 to 215 cells per condition) or H_2-DCFDA (30 to 47 cells for each condition). Bars depict average +SEM. Student's t-test revealed significant differences between the vehicle control and AuNC-treated cells (***, $p<0.001$), or between cells treated with 1 µM $Au_{15}SG_{13}$ and 1 µM $Au_{15}PEG$. Note that CellRox® Green and H_2-DCFDA differ in the signal-to-noise ratio and stability.

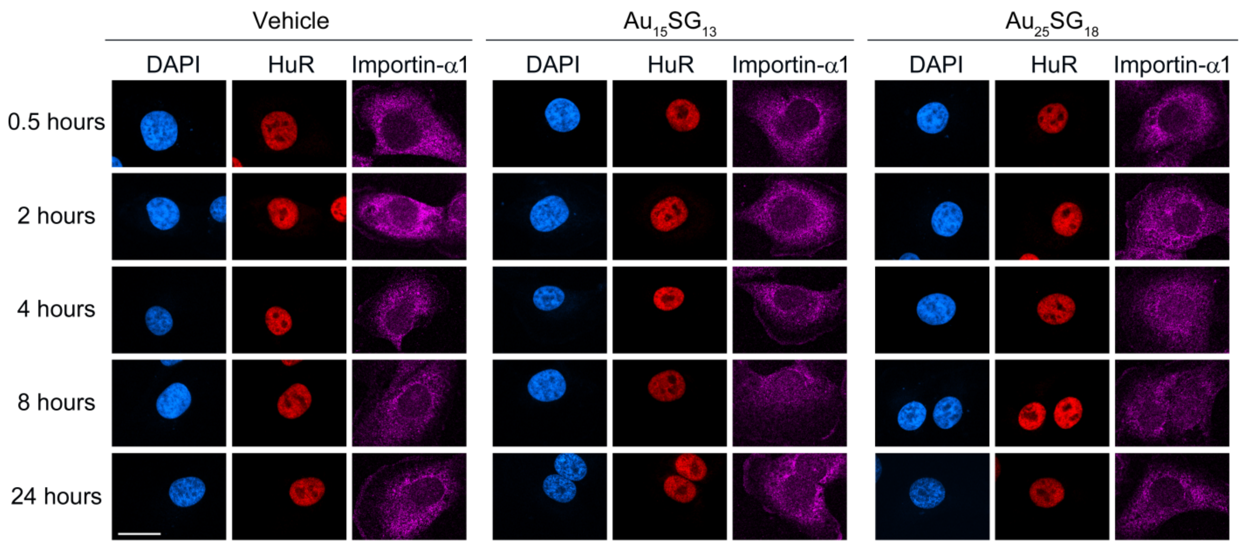

Figure S10. Lack of stress granule formation in response to AuNC treatment.

U251N cells were incubated with vehicle, 10 µM $Au_{15}SG_{13}$ or 10 µM $Au_{15}PEG$. At the times indicated, samples were fixed and processed for immunocytochemistry for the detection of HuR and importin-α1. Nuclei were stained with DAPI. Scale bar is 20 µm.

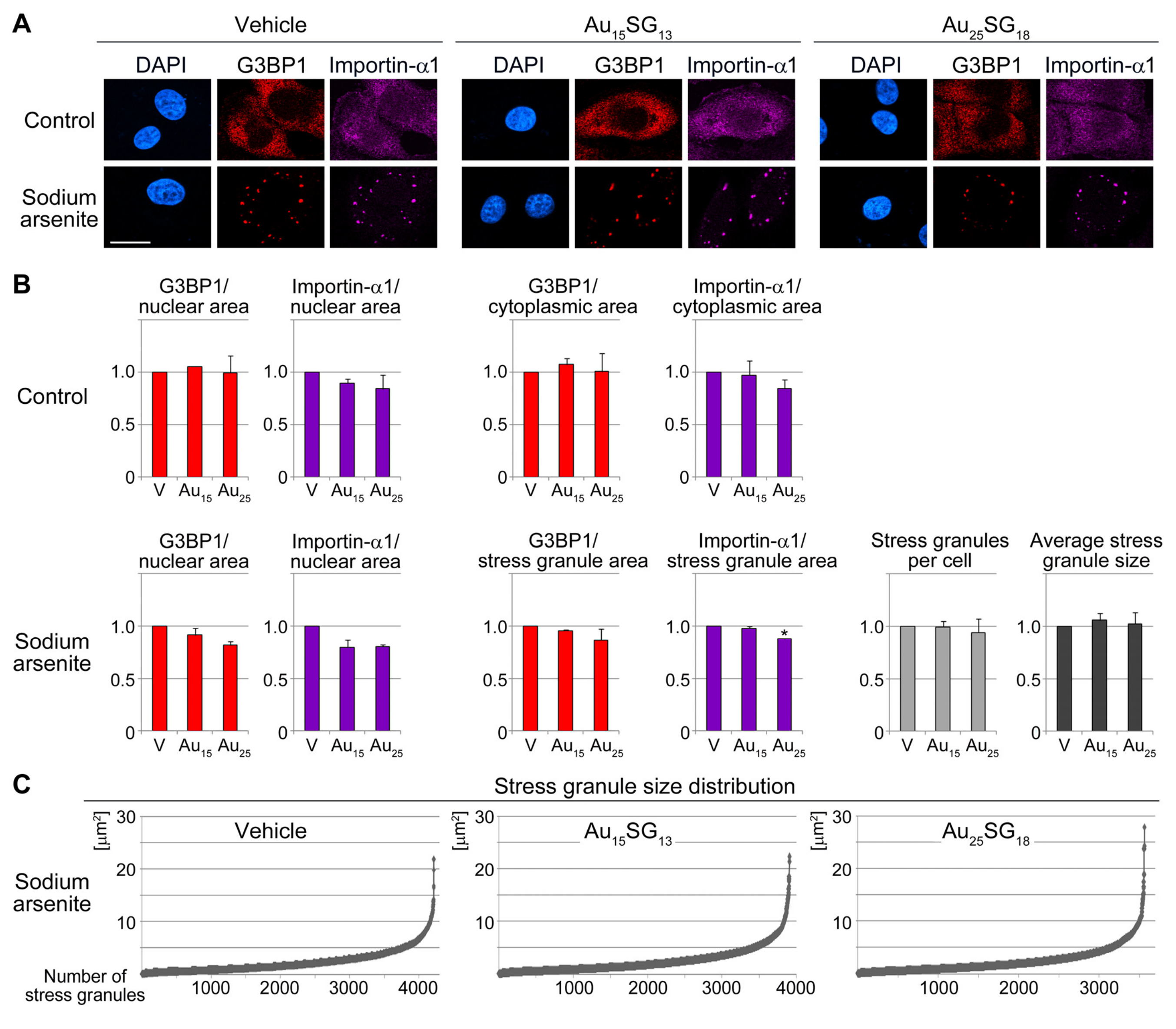

Figure S11. AuNC-induced changes in stress granule formation.

U251N cells were incubated with vehicle, 10 µM $Au_{15}SG_{13}$ (Au_{15}) or 10 µM $Au_{25}SG_{18}$ (Au_{25}) for 24 hours and kept under non-stress control conditions (control) or exposed to oxidative stress (0.5 mM sodium arsenite, 2 hours). (A) The stress granule components G3BP1 and importin-α1 were detected by immunocytochemistry. Nuclei were demarcated with DAPI; scale bar is 20 µm. (B) Fluorescence intensities were measured for G3BP1 and importin-α1. Results were normalized to vehicle controls (V). Fluorescence signals/area were quantified in the nuclear and cytoplasmic compartments for 162 to 203 non-stressed cells (control, no sodium arsenite) per experiment and for each treatment. Nuclear pixel intensities/area were determined for 112 to 149 arsenite-treated cells for each of two independent experiments and per condition. Between 1448 and 2422 stress granules were assessed per experiment for each treatment. One-way ANOVA combined with Bonferroni posthoc analysis was performed for statistical evaluation; *p<0.05. (C) The stress granule size distribution was determined for the combined results of two independent experiments.
